# Coixol ameliorates dopaminergic neurodegeneration by inhibiting neuroinflammation and protecting mitochondrial function

**DOI:** 10.3389/fphar.2025.1657910

**Published:** 2025-10-02

**Authors:** Shuo Yang, Jiaxi Han, Miao Xue, Yiming Zhang, Aohan Yan, Xiyu Gao, Jiangmei He, Xiaojia Sun, Shoupeng Fu, Dianfeng Liu, Bingxu Huang

**Affiliations:** ^1^ State Key Laboratory for Diagnosis and Treatment of Severe Zoonotic Infectious Diseases, Key Laboratory for Zoonosis Research of the Ministry of Education, Institute of Zoonosis, College of Veterinary Medicine, Jilin University, Changchun, China; ^2^ Department of Laboratory Animals, College of Animal Sciences, Jilin Provincial Key Laboratory of Animal Model, Jilin University, Changchun, Jilin, China; ^3^ Chongqing Research Institute, Jilin University, Chongqing, China

**Keywords:** coixol, neuroinflammation, NLRP3, mitochondrial dysfunction, Parkinson’s disease

## Abstract

Extensive research has revealed that neuroinflammation plays an important role in Parkinson’s disease (PD). Coixol, extracted from *Coix lacryma-jobi L.*, exhibits anti-inflammatory and antioxidant effects in various diseases. However, the effect of coixol on PD remains unclear. The aim of this study is to investigate the effects of coixol in a 1-methyl-4-phenyl-1,2,3,6-tetrahydropyridine (MPTP)-induced PD mouse model. Our results show that coixol improves the motor dysfunction and neuronal damage in PD mice by inhibiting neuroinflammation and maintaining mitochondrial function. Moreover, coixol suppressed the overactivation of the nuclear transcription factor κB (NF-κB) and the mitogen-activated protein kinase (MAPK) signaling pathways and regulated the NLR family pyrin structural domain 3 (NLRP3)/cysteinyl aspartate-specific proteinase-1 (Caspase1)/interleukin-1β (IL-1β) signaling pathway to inhibit neuroinflammation in PD mice. The results show that coixol mitigated reactive oxygen species (ROS)-induced mitochondrial damage, thereby inhibiting the overactivation of the NLRP3 inflammasome. Taken together, we found that coixol alleviates dopaminergic neurodegeneration in PD mice by inhibiting the activation of NF-κB and MAPK signaling pathways to suppress neuroinflammation and protect mitochondrial function from ROS production to regulate NLRP3 inflammasome activation.

## 1 Introduction

Parkinson’s disease (PD), an increasingly prevalent neurodegenerative disease affecting the elderly, has become a significant threat to their physical and mental health and brought a huge burden on families and socio-economic development (Di Napoli et al., 2024; Faouzi et al., 2023). Patients with PD suffer from characteristic motor symptoms, including resting tremor, bradykinesia, myotonia, and postural balance disorders (Huhn et al., 2024). The main pathological feature of PD is the progressive degeneration of dopaminergic neurons (DAs) in the substantia nigra (SN) of the midbrain and the striatum (Str), which governs the body’s motor function (Kouli et al., 2018). The precise mechanism underlying dopaminergic neurodegeneration remains elusive. Abundant evidence suggests that microglia play a pivotal role as the foremost defense against neuroinflammation in the brain (Harackiewicz and Grembecka, 2024; Yang et al., 2024; Bano et al., 2024). However, the clear mechanism linking microglia to dopaminergic neurodegeneration remains to be elucidated. It has been demonstrated that neuroinflammation, mitochondrial dysfunction, and oxidative stress contribute to the degeneration of DAs in the SN (Huang et al., 2022). Extensive research has reported that neuroinflammation derived from microglia plays a pivotal role in the pathogenesis of PD (Isik et al., 2023; Wu et al., 2021; Machado et al., 2016). In the early stages of the PD onset, activated microglia aggregate at the site of injury to regulate inflammatory cascades and facilitate the clearance of compromised cells or deleterious agents (Colonna and Butovsky, 2017). Overactivated microglia released substantial quantities of pro-inflammatory factors, resulting in neuronal degeneration and death, which further exacerbates the pathological process of PD.

The NLR family pyrin structural domain 3 (NLRP3) inflammasome participates in the regulation of inflammatory response and the pathogenesis of diseases (Jo et al., 2016). It has been reported that the activation of the NLRP3 inflammasome contributes to neuroinflammation in the central nervous system (Hung et al., 2020; Shi et al., 2020). A previous study has shown that activated NLRP3 inflammasome occurs in the brain tissue of patients with PD (Wang X. et al., 2020). The activated NLRP3 inflammasome enhances the expression of NLRP3 and activates its downstream signaling pathway to regulate neuroinflammation in the brain (Shao et al., 2016). Nuclear transcription factor κB (NF-κB), a key transcriptional activator, translocates to the nucleus, where it binds to pertinent DNA sequences to modulate the expression of inflammatory genes, such as NLRP3 (Li et al., 2022; Zhong et al., 2016). It has been reported that interactions between NF-κB and mitogen-activated protein kinase (MAPK) signaling pathways activate the NLRP3 inflammasome to regulate the inflammatory response in the pathogenesis of disease (Wang et al., 2023). The NLRP3 inflammasome, a pivotal protein hub orchestrating cellular pyroptosis, regulates neuroinflammation, which accelerates dopaminergic neurodegeneration in the pathogenic progression of PD (Zheng et al., 2023).

Oxidative stress and mitochondrial dysfunction are key pathophysiological processes in PD (Subramaniam et al., 2013). However, the intricate interplay between compromised mitochondrial function and neuroinflammation remains inadequately elucidated. NLRP3 inflammasome activation can be triggered by diverse intracellular signals, including reactive oxygen species (ROS) (Han et al., 2018). Moreover, it has been demonstrated that damaged mitochondria enhance the pro-inflammatory cascade response mediated by the NLRP3 inflammasome in microglia (Rawat et al., 2019). Extensive evidence has indicated that mitochondrial damage significantly affects the homeostasis and function of mitochondria in the brain (Ci et al., 2023; Wang W. et al., 2020; Baek et al., 2014). Damaged mitochondria can result in the excessive production of ROS, resulting in NLRP3 inflammasome activation (Dan Dunn et al., 2015). Maintaining appropriate intracellular ROS levels is essential for maintaining redox homeostasis and cell proliferation. Excessive ROS production impairs the activity of mitochondrial complex I, and mitochondrial dysfunction initiates oxidative stress and apoptosis, leading to the death of DAs and the clinical symptoms of PD (Dias et al., 2013). Therefore, inhibiting ROS production to alleviate mitochondrial dysfunction represents an effective strategy for regulating NLRP3 inflammasome-mediated neuroinflammation in PD. Previous research has revealed that damaged mitochondria result in an inadequate energy supply in the neurons of patients with PD (Gonzalez-Casacuberta et al., 2019). Therefore, maintaining the stability and functional integrity of mitochondria may improve mitochondrial energy metabolism and sustain energy production. The decrease in oxidative stress could alleviate mitochondrial dysfunction to inhibit neuroinflammation.

The treatment of PD remains limited, and there is currently no ideal treatment measure to alleviate its pathological process. Moreover, it has been the main aim of scientific research to find and develop a highly effective and non-toxic drug to relieve the symptoms of PD. *Coix lacryma-jobi L*, a food crop with high nutritional value, is often used to make porridge, cakes, wine, etc. Coixol, extracted from *C. lacryma-jobi L*, has been found to improve mouse models of lung injury (Shen et al., 2023) and liver injury (Zhou et al., 2024). Coixol downregulates the pro-inflammatory mediators, including interleukin-1β (IL-1β), interleukin-6 (IL-6), interleukin-18 (IL-18), tumor necrosis factor-α (TNF-α), nitrogen monoxide (NO), inducible nitric oxide synthase (iNOS), and cyclooxygenase-2 (COX-2) in activated macrophages (Hu et al., 2020), suggesting that coixol exerts significant anti-inflammatory activity. Coixol also alleviates oxidative and inflammatory responses by decreasing ROS production and increasing the activity of glutathione peroxidase (GSH-Px), glutathione reductase, and catalase (CAT). It has been reported that coixol modulates AChE activity, reduces oxidative stress, and inhibits Aβ aggregation in both *in vitro* and *in silico* studies, suggesting that coixol holds promise as a lead compound for developing novel therapeutic strategies to manage Alzheimer’s disease and related neurodegenerative disorders (Amen et al., 2025). These findings indicate that coixol possesses high antioxidant activity. However, the potential role and mechanism of coixol in PD remain unclear. In this study, we investigated the effect of coixol on neuroinflammation in LPS-treated microglia and in a 1-methyl-4-phenyl-1,2,3,6-tetrahydropyridine (MPTP)-induced mouse PD model. We investigated whether coixol exerts a neuroprotective effect on PD mice by inhibiting ROS production to protect mitochondrial function in microglia.

## 2 Materials and methods

### 2.1 Chemical compounds and reagents

Coixol (purity ≥98%) was supplied by Yuanye (Shanghai, China), and LPS was obtained from Shanghai Aladdin Biochemical Technology Co., Ltd. MPTP (purity ≥98%) and MPP+ (purity ≥98%) were obtained from Merck (Darmstadt, Germany). NAC (an ROS inhibitor) was obtained from Beyotime, Shanghai, China. Fetal bovine serum (FBS) was supplied by HyClone (Logan, Utah, United States). TRIzol reagent was purchased from BestBio, Shanghai, China. Sodium carboxymethylcellulose (CMC) was obtained from Macklin (Shanghai, China). NP40, Dulbecco’s modified Eagle’s medium (DMEM), 0.05% trypsin, 0.25% trypsin, and dimethyl sulfoxide (DMSO) were purchased from ServiceBio (Wuhan, China).

### 2.2 PD mouse model and treatment

Thirty male C57BL/6 mice (8–10 weeks old) were purchased from Liaoning Changsheng Biotechnology Co., Ltd. (Benxi, China). The mice were kept on a 12 h/12 h light/dark cycle and had ad libitum access to food and water. After acclimation, the mice were randomly assigned to five groups: the non-treated (NT) group, the MPTP group, the coixol (100 mg/kg/d) group, and the coixol (50 or 100 mg/kg/day) + MPTP group. The mice received intragastric administration of coixol for 24 days. They were intraperitoneally injected with MPTP at a dose of 30 mg/kg/day for 7 days to establish the PD mouse model. In the coixol + MPTP group, mice received intragastric coixol for 3 days prior to MPTP treatment and continued for 14 days after MPTP injection. NT mice received intragastric saline treatment.

All experiments were conducted in strict accordance with GB1492S. The animal experiments in this study were performed according to the guidelines of the Animal Welfare Committee (IACUC) of Jilin University (protocol no. SY202404021) and complied with the ethical requirements for the use of experimental animals. In this study, every effort was made to minimize the number of animals used and reduce pain.

### 2.3 Cell culture

Mouse microglia BV-2 cells and mouse dopaminergic neuron SN4741 cells were obtained from Shanghai Bin Sui Biotechnology (Shanghai, China). The cells were cultured in 90% DMEM + 10% FBS under an atmosphere of 95% air and 5% carbon dioxide at 37 °C. When the density of cells reached 80%, the cells were digested and passaged with 0.25% trypsin in SN4741 cells or 0.05% trypsin in BV-2 cells.

### 2.4 Behavioral testing

#### 2.4.1 Open-field test

To assess locomotor activity and exploratory behavior, the open-field (OF) test was conducted using four plastic open-field chambers, each measuring 50 × 50 × 40 cm, ensuring a serene environment. Before each test, the chamber was cleaned and made odor-free using alcohol to prevent interference with subsequent experiments. After acclimatization to the chamber for 3 days, the mice were placed in the center and allowed to move freely for 5 min, with their movement trajectories automatically recorded. The experiment was conducted at the same time of day under uniform lighting conditions.

#### 2.4.2 Pole test

After the mice were trained for 3 days, the pole test was conducted. The wooden pole used was 50 cm long, and a ball was attached to its top. Mice were placed on the ball at the beginning of the test. The time it took the mice to climb down the pole was recorded, and the test was repeated if a mouse stopped in the middle or climbed in the opposite direction. Each test was repeated three times, and the average time was recorded.

#### 2.4.3 Rotating rod test

The mice were acclimated to the rotarod fatigue device for 3 days prior to the test. They were placed on the rotarod in the center of the roller during the test. When the power was switched on and the speed was set, the roller rotated automatically, and the rotating lever gradually increased the speed from 10 to 50 rpm per minute over 2 min. The mice were acclimated to the rotarod fatigue device and then underwent three consecutive trials of 2 min each. The time spent by the mice on the rotating rod was recorded. Each test was repeated three times, and the mean value was calculated.

### 2.5 RT-PCR assay

RNA was extracted from cells and midbrain tissue using triazole, chloroform, and isopropanol. cDNA was then reverse-transcribed using a reverse transcription kit to provide a template for subsequent RT-qPCR and Primer sequence in Table 1. The cDNA was then amplified, and the Cq values were recorded using the Bio-Rad system. The relative amount of mRNA to β-actin in the medium was determined using the value of 2^−ΔΔCT^.

**TABLE 1:** Base sequence of each gene.

RT-qPCR	5′ to 3′
*IL-6-F*	GAC​AAA​GCC​AGA​GTC​CTT​CAG​A
*IL-6-R*	TGT​GAC​TCC​AGC​TTA​TCT​CTT​GG
*IL-1β-F*	CCA​CAG​ACC​TTC​CAG​GAG​AAT​G
*IL-1β-R*	GTG​CAG​TTC​AGT​GAT​CGT​ACA​GG
*IL-18-F*	GAT​AGC​CAG​CCT​AGA​GGT​ATG​G
*IL-18-R*	CCT​TGA​TGT​TAT​CAG​GAG​GAT​TCA
*TNF-α-F*	CAG​GCG​GTG​CCT​ATG​TCT​C
*TNF-α-R*	CGA​TCA​CCC​CGA​AGT​TCA​GTA​G
*iNOS-F*	ACA​TCG​ACC​CGT​CCA​CAG​TAT
*iNOS-R*	CAG​AGG​GGT​AGG​CTT​GTC​TC
*COX2-F*	TGC​ACT​ATG​GTT​ACA​AAA​GCT​GG
*COX2-R*	TCA​GGA​AGC​TCC​TTA​TTT​CCC​TT
*β-actin-F*	GGC​TGT​ATT​CCC​CTC​CAT​CG
*β-actin-R*	CCA​GTT​GGT​AA-CAA​TGC​CAT​GT

### 2.6 Immunohistochemical staining

After the treatment, the mice were euthanized, and their midbrains were immersed in 4% paraformaldehyde for 36 h. The fixed midbrain tissues of PD mice were successively placed in 70%, 80%, 90%, and 95% ethanol solution for 2 h, then in anhydrous ethanol for 2 h, after which they were dehydrated twice, soaked in xylene for 15 min, cleared twice, embedded in paraffin wax three times at 60 °C for 30 min each time, and cooled at 4 °C. Afterward, the tissues were cut into 6 µm slices each and dried at 75 °C. Immunohistochemical (IHC) staining was then performed using the Anti-Mouse/Rabbit Universal Immunohistochemistry Test Kit (Proteintech, Wuhan, China), according to the manufacturer’s specifications. Dopaminergic neurons were labeled with the anti-tyrosine hydroxylase (TH) antibody (1:250, Proteintech), and microglia were labeled with the anti-ionized calcium-binding adaptor molecule 1 (IBA-1) antibody (1:1,000, Proteintech). The results were analyzed using ImageJ software.

### 2.7 Cell viability assay

The cells were inoculated into 96-well plates at a density of 1 × 10^5^ cells/well. When the density was moderate, coixol (50 μM or 100 μM) was added to treat the cells. After 24 h, the cell culture medium was discarded, the plates were washed with phosphate buffer three times, and 100 μL of CCK-8 dilution was added to each well, which were then cultured in an incubator for 2 h. The absorbance of the cells was detected using an enzyme marker at 450 nm, and the proliferative activity of the cells was calculated and analyzed. The experiment was repeated three times.

### 2.8 LDH assay

Cells were seeded in 96-well plates at a density of 3 × 10^4^ per well. When the cells had grown to approximately 70% confluence, they were treated with different stimuli. After the cells were treated for 24 h, the amount of LDH released into the medium was investigated using an LDH assay kit according to the manufacturer’s instructions.

### 2.9 Western blot assay

The experimental protocol was followed for the treatment of BV-2 cells, SN4741 cells, and C57BL/6 mice. The cells and SN tissue of the mice were harvested and lysed with NP-40. Protein concentrations were then determined using the BCA Protein Assay Kit (Thermo Fisher Scientific, Rockford, IL, United States). The SDS-PAGE electrophoresis sample volume for each sample was calculated based on protein concentration. The samples were mixed with 5× sample buffer at a ratio of 4:1 and then loaded onto a 15% SDS-PAGE gel, which was used for the separation of the protein samples. Following electrophoresis, the gel proteins were transferred onto ethanol-activated PVDF membranes using the semi-dry membrane transfer method at a regulated voltage of 80 V for 80 min. Subsequently, the PVDF membrane was sealed on a shaker with 5% skim milk for a period of 2 h. Following this, the membrane was washed thoroughly with TBST, after which the primary antibodies GFAP (1:5,000) and p38 (1:1,000) were provided by Cell Signaling Technology (Danvers, MA, United States); cysteine-requiring aspartate protease 3 (Caspase3, 1:1,000), *B-cell leukemia/lymphoma* 2 (Bcl-2, 1:1,000), Bcl-2-associated X protein (Bax, 1:1,000), NLRP3 (1:1,000), cysteinyl aspartate specific proteinase-1 (Caspase-1, 1:1,000), IL-1β (1:1,000), gasdermin D (GSDMD, 1:1,000), gasdermin-N domains in GSDMD (GSDMD-N, 1:1,000), apoptosis-associated speck-like protein containing a CARD (ASC, 1:1,000), silent information regulator 1 (Sirt1, 1:800), silent information regulator 5 (Sirt5, 1:1,000), peroxisome proliferator-activated receptor γ coactivator l alpha (PGC-1α, 1:800), NAD (P) H quinone dehydrogenase 1 (NQO1, 1:500), mitofusin 1 (MFN1, 1:500), mitofusin 2 (MFN2, 1:500), translocase of outer mitochondrial membrane 20 (TOM20, 1:1,200), fun14 domain-containing protein 1 (FUNDC1, 1:2,000), TH (1:2,000), phospho-JNK1/2 (p-JNK; 1:3,000), NF-κB p65 (p65, 1:1,000), phospho-IκB (p-IκB, 1:2,000), COX2 (1:2,500), and iNOS (1:4,000) were purchased from Proteintech (Wuhan, China); phospho-NF-κB p65 (p-p65, 1:4,000), IκB (1:2,000), phospho-ERK1/2 (p-ERK, 1:2,000), ERK1/2 (1:2,000), phospho-p38 (p-p38, 1:1,000), JNK1/2 (1:4,000), and β-actin (1:5,000) were purchased from Abcam (Cambridge, United Kingdom).) and incubated at 4 °C overnight. The membrane was washed five times for 10 min each in TBST and then incubated with the secondary antibody (1:5,000) for 2 h at room temperature (RT). After being incubated, the blots were washed five times for 10 min each in TBST. Finally, the ECL chemiluminescent solution was added, and the protein bands were photographed using the Bio-Rad Gel Imaging Analyzer (Bio-Rad Laboratories, Hercules, CA, United States). The grayscale values of the proteins were then analyzed using ImageJ software.

### 2.10 Immunofluorescence staining

Immunofluorescence was divided into animal tissue immunofluorescence and cellular immunofluorescence. Immunofluorescence of animal tissues: mouse midbrains were collected according to the corresponding grouping; deparaffinized with xylene, repeated twice; hydrated with anhydrous ethanol 1, anhydrous ethanol 2, 95% alcohol, 90% alcohol, 80% alcohol, 70% alcohol, and distilled water for 5 min each; then permeabilized and antigen-retrieved with 0.1% Triton X-100; and blocked in fire-wool gel (15 min), PBST (10 min), and 5% donkey serum (1 h) for desiccation. Primary antibodies for NLRP3 and TH (1:300, Proteintech, Wuhan, China) were used for midbrain tissues. All sections were treated with PBS (5 min/time, three times) and then incubated with goat anti-rabbit IgG (1:2000; Proteintech, Ltd.) for 1 h at room temperature. After being treated with PBS (5 min/time, three times), the sections were incubated with 4′,6-diamidino-2-phenylindole (DAPI) in the dark.

Cell immunofluorescence: The primary steps of immunofluorescence experiments included cell slice preparation, fixation and permeabilization, blocking, antibody incubation, and fluorescence detection. The cells were washed three times with PBS, fixed with immunofluorescence fixative, permeabilized with 0.01% Triton X-100, blocked with 5% BSA, and incubated with the primary antibodies (p65 1:500, ASC 1:300, and NLRP3 1:300) overnight. The cells were then washed again and treated with a fluorescent secondary antibody for 1 hour. Finally, the nuclei were stained with DAPI. The results were observed under a confocal microscope.

### 2.11 Intracellular antioxidant indexes

CAT, SOD, MDA, adenosine triphosphate (ATP), and ROS levels in SN4741 cells and mouse serum samples were measured according to the manufacturer’s instructions.

### 2.12 Statistical analysis

Data are presented as the mean ± SEM and analyzed using GraphPad Prism 9.4 (GraphPad Software, United States). A Student’s t-test and one-way ANOVA were used for comparison between and among groups, respectively, followed by a Tukey’s *post hoc* test. Statistical significance was set at ^*^ *p* < 0.05, ^**^ *p* < 0.01, and ^***^ *p* < 0.001.

## 3 Results

### 3.1 Coixol ameliorates motor dysfunction in MPTP-injected PD mice

**FIGURE 1:**
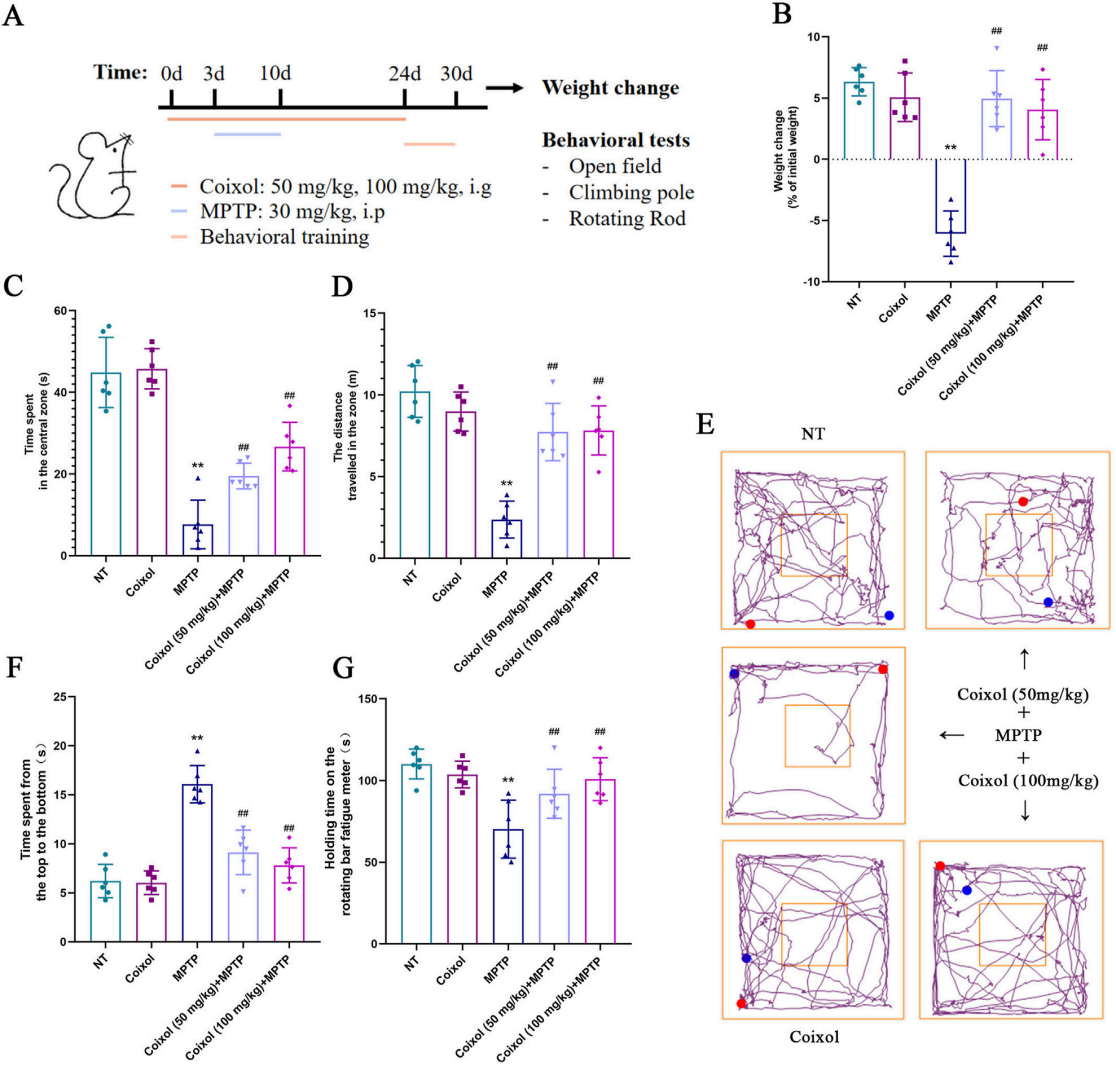
Effects of coixol on weight loss and motor dysfunction were investigated in 1-methyl-4-phenyl-1,2,3,6-tetrahydropyridine (MPTP)-treated mice. **(A)** Schematic representation of the experimental design. **(B)** Weight changes in mice were recorded. **(C)** Time spent by the mice in the central zone was measured. **(D)** Total distance traveled by the mice was investigated using the open-field test. **(E)** The trajectories of the mice in the open-field test were recorded. **(F)** The time it took the mice to descend from the top to the bottom was recorded. **(G)** The time spent on the rotating rod by the mice was recorded. Data are presented as the mean ± SEM (n = 6). ^**^ *p < 0.01* vs. NT group. ^*##*^ *p < 0.01* vs. MPTP-treated group.

To investigate whether coixol exerts a protective effect on motor dysfunction in a mouse model of PD, we established a PD mouse model using MPTP over 7 days. After MPTP injection, the mice received intragastric administration of coixol for 2 weeks (Figure 1A). Our results showed that the weight loss was significant, and the pretreatment with coixol decreased the rate of weight loss (Figure 1B). Then, we detected the locomotor ability of the mice by performing the open-field test, rotating rod test, and pole test. The spontaneous activity, exploratory behavior, and anxiety and depression states of the mice were evaluated using the total distance moved in the open field. The locomotor ability, coordination, and crawling ability of the mice were assessed using the time spent in the pole test. The coordinated locomotion, balance, and exercise endurance of the mice were investigated using the time spent in the rotating rod test. We found that the total locomotor distance was dramatically lower in the MPTP group than in the NT group. The locomotor distance and time spent in the center zone were higher in the coixol (50 mg/kg) + MPTP group and the coixol (100 mg/kg) + MPTP group than in the MPTP group (Figures 1C–E). The rod-climbing test showed that the MPTP group took significantly longer to complete the test compared with the NT group. The rod-climbing time of the mice in the coixol (100 mg/kg) + MPTP group was greatly improved compared with that in the MPTP group (Figure 1F). In the baton twirling test, the mice in the MPTP group spent significantly less time on the baton compared with the NT group, whereas the balance of the mice during baton twirling was significantly improved after coixol treatment (Figure 1G). These results reveal that coixol improves MPTP-induced weight loss and motor behavioral deficits in PD mice.

### 3.2 Coixol ameliorates dopaminergic neuronal damage in MPTP-injected mice

**FIGURE 2:**
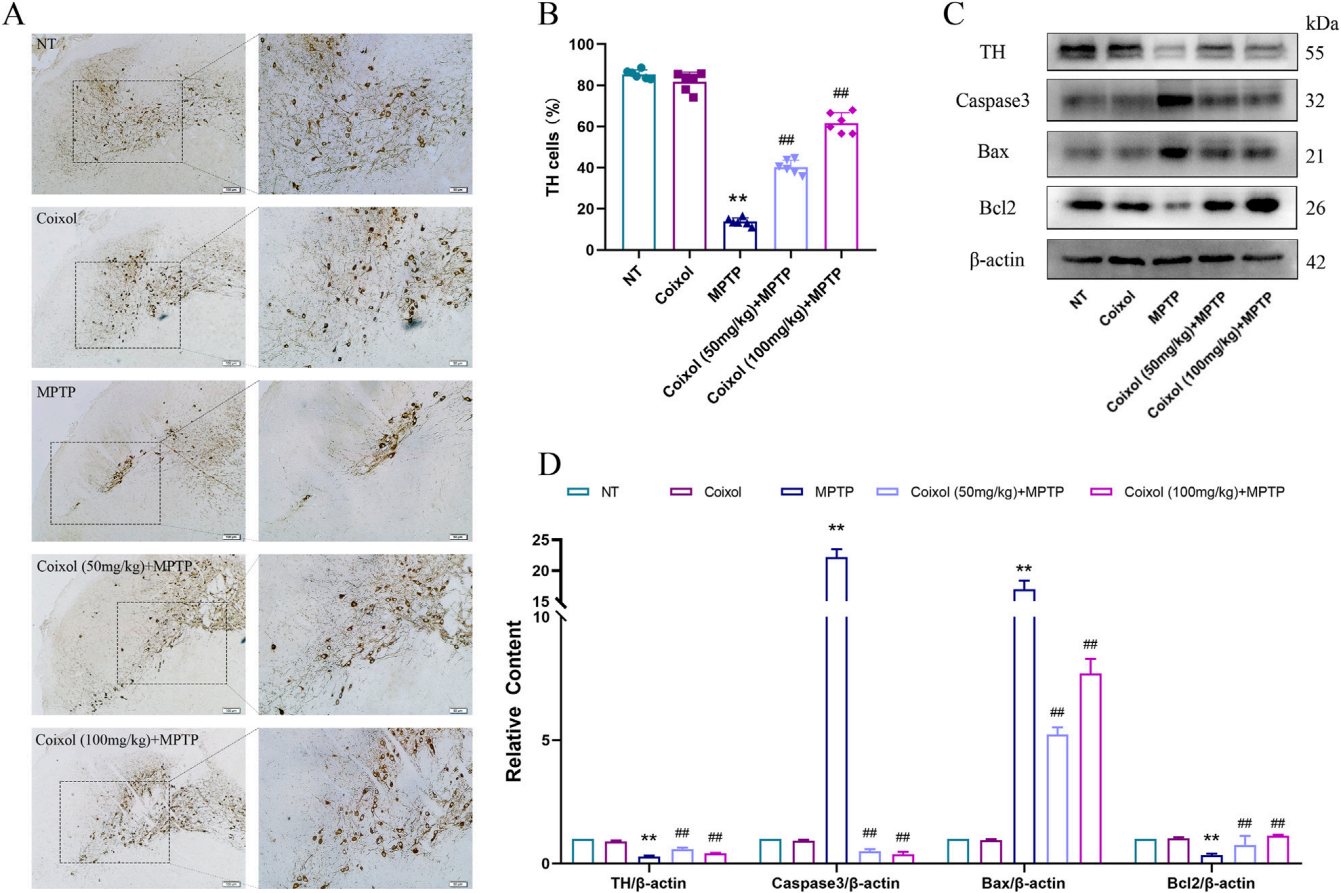
Effect of coixol on dopaminergic neurons was measured in the substantia nigra (SN) of MPTP-induced PD mice. **(A,B)** Tyrosine hydroxylase (TH)-positive cells (scale bar = 250 μm) in the SN were measured using immunohistochemistry (IHC). **(C,D)** The expressions of TH, cysteine-requiring aspartate protease 3 (Caspase3), *B-cell leukemia/lymphoma* 2 (Bcl2), and Bcl2-associated X protein (Bax) were measured using Western blot analysis in the SN of PD mice. Results are presented as the mean ± SEM (n = 3). ^**^ *p < 0.01* vs. NT group. ^*##*^ *p < 0.01* vs. MPTP-treated group.

To further investigate the neuroprotective effects of coixol, we investigated the expression of DA through immunohistochemical staining and Western blotting. Our results showed that MPTP treatment decreased TH expression in the midbrain SN of mice. The expression of TH was increased in the coixol + MPTP group compared with that in the MPTP group (Figures 2A,B). Western blotting confirmed the immunohistochemical results (Figures 2C,D). Meanwhile, the expressions of Caspase-3, Bcl-2, and Bax in the SN (Figures 2C,D) were quantitatively analyzed. The expressions of Caspase-3 and Bax in the midbrain were found to be significantly reduced, and the expression of Bcl-2 in the midbrain was increased (Figures 2C,D). These results suggest that coixol ameliorates DA damage in the midbrain of PD mice.

### 3.3 Inhibition of coixol on MPTP-induced microglia activation

**FIGURE 3:**
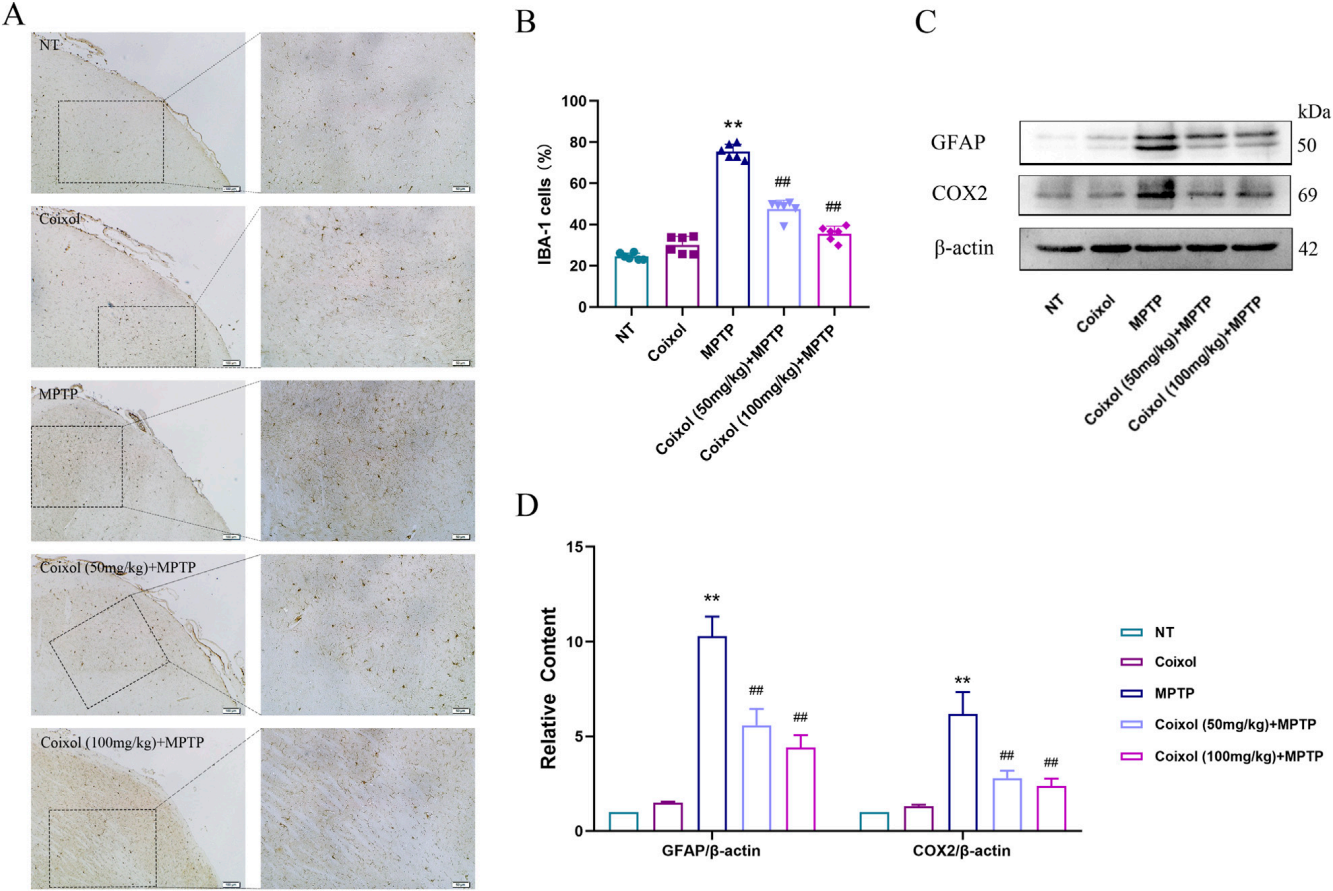
Inhibition of coixol on MPTP-induced microglia activation. **(A,B)** Ionized calcium-binding adaptor molecule 1 (IBA-1)-positive cells (scale bar = 100 μm) were stained in microglia using immunohistochemistry (IHC). **(C,D)** Levels of glial fibrillary acidic protein (GFAP) and cyclooxygenase-2 (COX2) were measured using Western blot analysis in the microglia. Results are presented as the mean ± SEM (n = 3). ^**^ *p < 0.01* vs. NT group. ^*##*^ *p < 0.01* vs. MPTP-treated group.

To investigate the effect of coixol on MPTP-induced microglia activation in the SN of PD mice, we detected microglia activation in the SN using immunohistochemistry, and the expressions of GFAP and COX-2 were detected using Western blotting. These results showed that MPTP treatment increased the expression of GFAP in the SN. The expression level of GFAP was significantly decreased in the coixol + MPTP group compared with that in the MPTP group (Figures 3A,B). Western blotting results showed that coixol treatment inhibits microglia activation in PD mice (Figures 3C,D). The results demonstrate that coixol inhibits microglia activation, thereby exerting a neuroprotective effect in PD mice.

### 3.4 Coixol inhibits NLRP3 inflammasome activation in the SN of PD mouse midbrains

**FIGURE 4:**
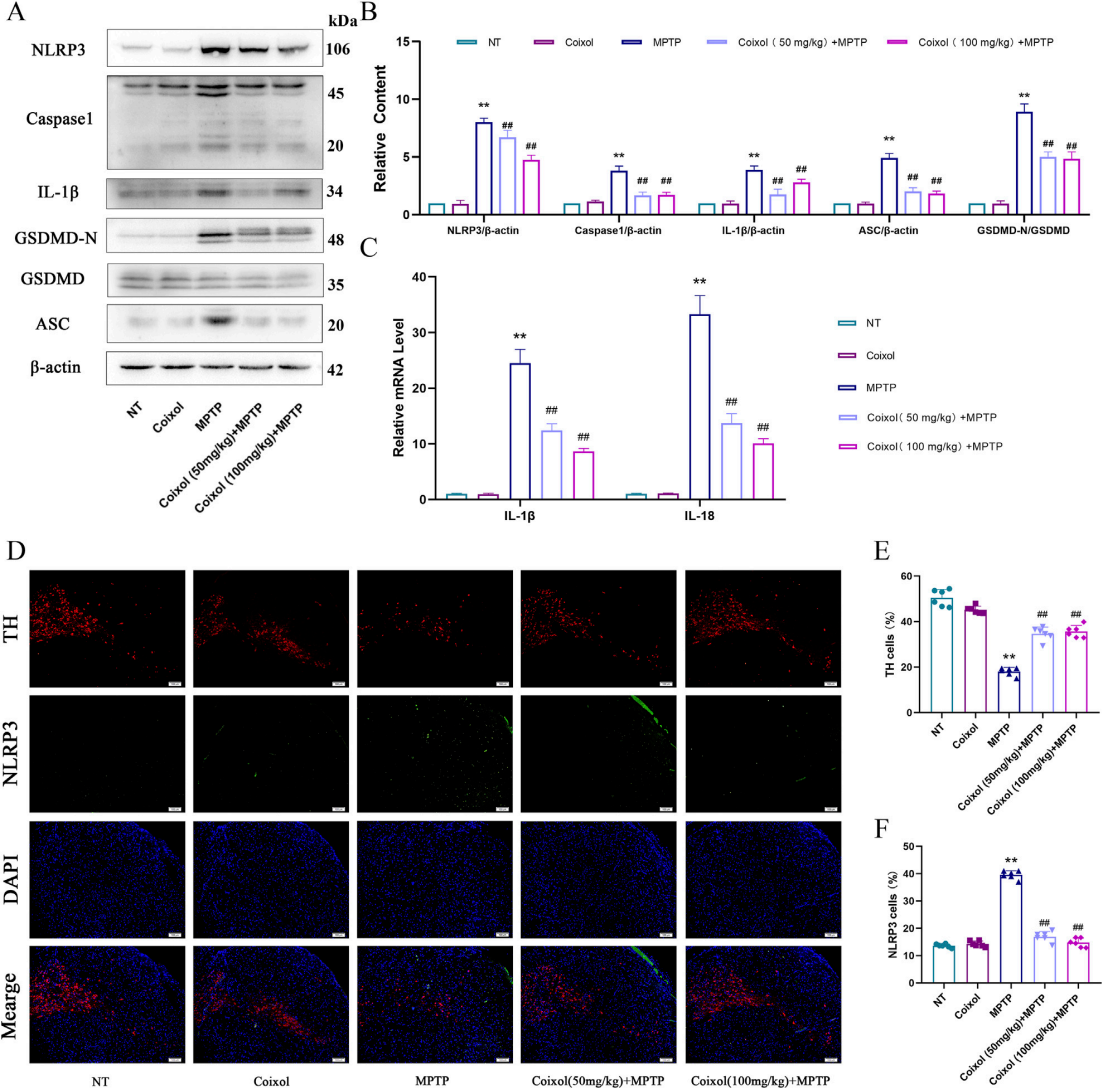
Effect of coixol on NLRP3 inflammasome activation in the SN of MPTP-induced PD mice. **(A,B)** The protein expressions of NOD-like receptor thermal protein domain-associated protein 3 (NLRP3), cysteinyl aspartate-specific proteinase-1 (Caspase-1), interleukin-1β (IL-1β), gasdermin D (GSDMD), gasdermin-N domains in GSDMD (GSDMD-N), and apoptosis-associated speck-like protein containing a CARD (ASC) in the SN were measured using Western blot analysis. **(C)** mRNA expressions of interleukin-1β (IL-1β) and interleukin-18 (IL-18) in the SN. **(D,E)** Immunofluorescence of TH (red) and DAPI (blue) in the SN. **(D,F)** Immunofluorescence of NLRP3 (green) and DAPI (blue) in the SN. Results are presented as the mean ± SEM (n = 3). ^**^ *p* < *0.01* vs. NT group. ^*##*^ *p* < *0.01* vs. MPTP-treated group.

Microglia activation increases the production of pro-inflammatory mediators, and NLRP3 inflammasome activation exacerbates the inflammatory response. To investigate the effect of coixol on NLRP3 inflammasome activation in PD mice, the expression levels of NLRP3, Caspase-1, IL-1β, ASC, and GSDMD-N were examined in the midbrain of MPTP-induced mice through Western blot analysis (Figures 4A,B). These results showed that the expressions of NLRP3, Caspase-1, IL-1β, ASC, and GSDMD-N were significantly upregulated in PD mice, indicating that the NLRP3 inflammasome was activated. Coixol treatment significantly inhibited the activation of NLRP3 and the expression of the inflammatory factors IL-1β and IL-18 in PD mice (Figure 4C). Meanwhile, immunofluorescence staining results showed that the expression of TH was decreased and the expression of NLRP3 was increased in the MPTP-treated group (Figures 4D–F). Meanwhile, coixol treatment increased the expression of TH and weakened the expression of NLRP3, as determined by immunofluorescence staining. These results indicate that coixol inhibits MPTP-induced NLRP3 inflammasome activation in the SN region of PD mice.

### 3.5 Coixol suppresses the inflammatory response in LPS-induced BV-2 cells

**FIGURE 5:**
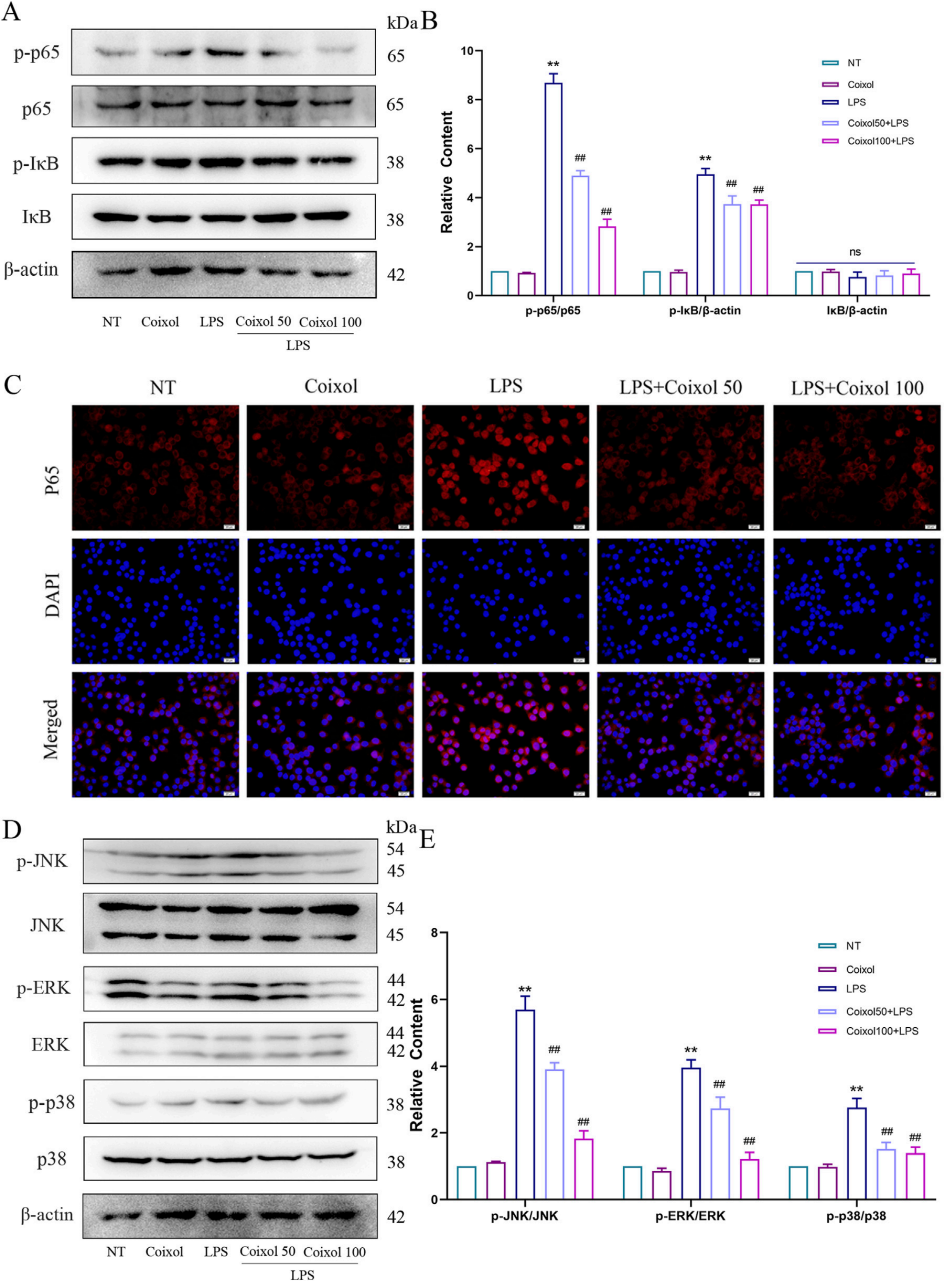
Effects of coixol on NF-κB and MAPK signaling pathways. **(A,B)** The protein levels of p-p65, p65, p-IκB, and IκB were measured in LPS-induced BV-2 cells using Western blot analysis. **(C)** Immunofluorescence staining of p65 (red) and DAPI (blue) in BV-2 cells. **(D,E)** The protein levels of p-JNK, JNK, p-p38, p38, p-ERK, and ERK were measured in LPS-induced BV-2 cells using Western blot analysis. Results are presented as the mean ± SEM (n = 3). ^*ns*^ *p>0.05*, ^**^ *p* < *0.01* vs. NT group. ^*ns*^ *p>0.05,* and ^*##*^ *p* < *0.01* vs. LPS-treated group.

To investigate whether coixol inhibits the inflammatory response, we detected the expressions of iNOS and COX2 in LPS-treated BV-2 cells using a Western blot assay. The results showed that coixol significantly inhibited the protein and mRNA expressions of iNOS and COX2 in LPS-induced BV-2 cells (Supplementary Figure S1). To further investigate the anti-inflammatory mechanism of coixol, we investigated the effect of coixol on NF-κB and MAPK signaling pathways in LPS-treated BV-2 cells (Figures 5A,D). The results showed that the expressions of p-p65 (Figures 5A,B), p-IκB (Figures 5A,B), p-JNK (Figures 5D,E), p-ERK (Figures 5D,E), and p-p38 (Figures 5D,E) were significantly increased compared with those in the NT group. Coixol treatment suppressed the inflammatory response in LPS-treated BV-2 cells in a dose-dependent manner. Moreover, the nuclear translocation of p65 was detected in LPS-treated BV-2 cells through cellular immunofluorescence staining. The results show that coixol significantly inhibits the nuclear translocation of p65 (Figure 5C). In conclusion, coixol was found to inhibit the activation of NF-κB and MAPK signaling pathways to inhibit the inflammatory response in LPS-induced BV-2 cells.

### 3.6 Effect of coixol on NLRP3 inflammasome activation in BV-2 cells

**FIGURE 6:**
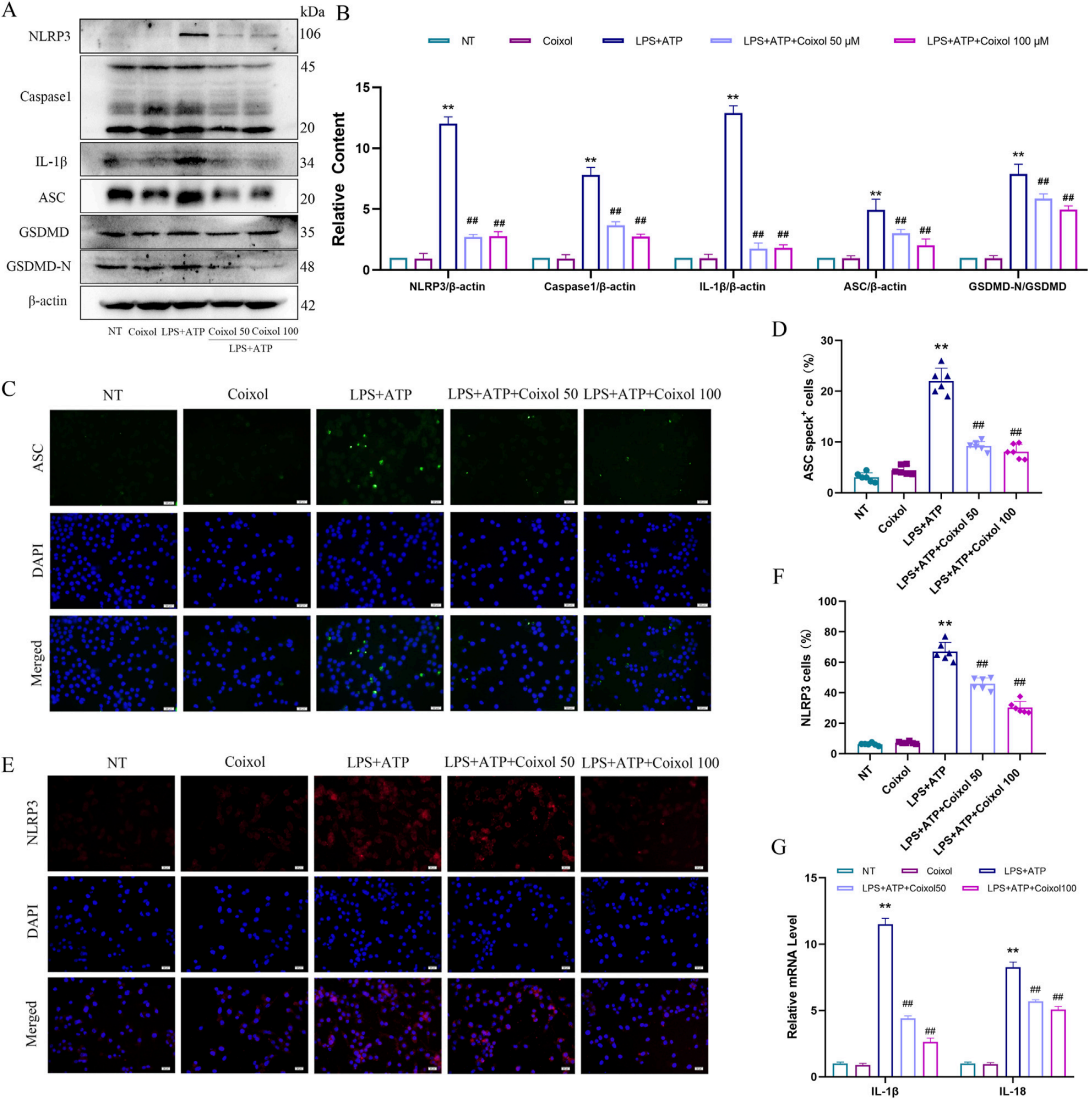
Effects of coixol on NLRP3 inflammasome activation. **(A,B)** The protein levels of NOD-like receptor thermal protein domain-associated protein 3 (NLRP3), cysteinyl aspartate-specific proteinase-1 (Caspase-1), interleukin-1β (IL-1β), apoptosis-associated speck-like protein containing a CARD (ASC), gasdermin D (GSDMD), and gasdermin-N domains in GSDMD (GSDMD-N) were investigated in LPS-induced BV-2 cells using Western blot analysis. **(C,D)** Immunofluorescence staining of ASC (green) and DAPI (blue) in BV-2 cells. **(E, F)** Immunofluorescence of NLRP3 (red) and DAPI (blue) in BV-2 cells. **(G)** The mRNA expressions of IL-1β and interleukin-18 (IL-18) in BV-2 cells. Results are presented as the mean ± SEM (n = 3). ^**^ *p* < *0.01* vs. NT group. ^*##*^ *p* < *0.01* vs. LPS-treated group.

Activated NLRP3 inflammasome were found to exacerbate the inflammatory response. To detect the effect of coixol on NLRP3 inflammasome, the expressions of NLRP3, Caspase-1, IL-1β, ASC, and GSDMD-N were measured (Figures 6A,B). Our results showed that treatment with LPS+ATP significantly upregulated the expressions of NLRP3, Caspase-1, IL-1β, ASC, and GSDMD-N, indicating that NLRP3 inflammasome were activated in BV-2 cells. Coixol treatment significantly inhibited NLRP3 inflammasome activation. The results of cellular immunofluorescence showed that the expressions of ASC (Figures 6C,D) and NLRP3 (Figures 6E,F) were increased in BV-2 cells treated with LPS+ATP. The results showed that the fluorescence intensities of both ASC and NLRP3 were weakened by the addition of a high dose of coixol (Figures 6E,F). We found that coixol pretreatment inhibited the expressions of inflammatory factors IL-1β and IL-18 in BV-2 cells (Figure 6G). These results indicate that coixol can inhibit the activation of the NLRP3 inflammasome.

### 3.7 Coixol protects mitochondrial function in MPP^+^-treated SN4741 cells by activating the Sirt1/Sirt5/PGC1-α/NQO1 signaling pathway

**FIGURE 7:**
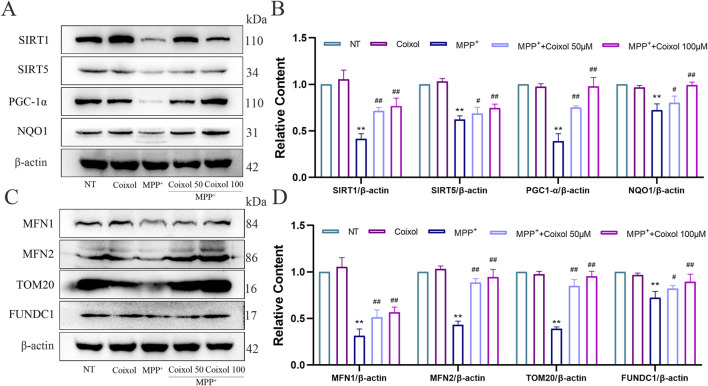
Effect of coixol on mitochondrial function in MPP^+^-induced SN4741 cells. **(A,B)** The protein levels of silent information regulator 1 (Sirt1), silent information regulator 5 (Sirt5), peroxisome proliferators-activated receptor γ coactivator l alpha (PGC-1α), and NAD (P) H quinone dehydrogenase 1 (NQO1) were measured in MPP^+^-induced SN4741 cells using Western blot analysis. **(C,D)** The protein levels of mitofusin 1 (MFN1), mitofusin 2 (MFN2), translocase of the outer mitochondrial membrane 20 (TOM20), and fun14 domain-containing protein 1 (FUNDC1) were investigated in MPP^+^-induced SN4741 cells using Western blot analysis. Results are presented as the mean ± SEM (n = 3). ^**^ *p* < *0.01* vs. NT group. ^*#*^ *p < 0.05 and* ^*##*^ *p* < *0.01* vs. MPP^+^-treated group.

The effect of coixol on ROS expression was also detected using an ROS analysis kit and cellular immunofluorescence staining (Supplementary Figures S2B,C). We detected the expression of mitochondrial membrane potential JC-1 using immunofluorescence staining. Our results revealed that the green fluorescent signal (JC-1 monomer) was enhanced and the red fluorescent signal (JC-1 aggregate) disappeared in the MPP+ group (Supplementary Figure S2D), suggesting a decrease in mitochondrial membrane potential in the MPP+-treated group. The effects of coixol on various oxidative damage indicators were further investigated in MPP^+^-induced SN4741 cells. The results showed that the levels of ATP, SOD, and CAT were significantly reduced, and MDA levels were increased in the MPP^+^ group compared with those in the NT group (Supplementary Figure S3). The Sirt1/Sirt5/PGC1-α/NQO1 signaling pathway is an important regulatory mechanism of intracellular mitochondrial energy metabolism. Therefore, we further investigated the effect of coixol on the Sirt1/Sirt5/PGC1-α/NQO1 signaling pathway in MPP^+^-induced SN4741 cells. The expression levels of Sirt1, Sirt5, PGC1-α, and NQO1 were significantly decreased compared with those in the NT group (Figures 7A,B). Western blotting results showed that the levels of mitochondrial fusion and division-related proteins (MFN1, MFN2, TOM20, and FUNDC1) were significantly decreased in MPP^+^-induced SN4741 cells compared with those in the NT group (Figures 7C,D). Coixol treatment significantly increased the expression levels of MFN1, MFN2, TOM20, and FUNDC1. The results indicate that coixol can increase the level of mitochondrial energy metabolism and enhance the balance between fusion and division of the outer mitochondrial membrane by activating the Sirt1/Sirt5/PGC1-α/NQO1 signaling pathway in MPP^+^-induced SN4741 cells.

### 3.8 Effect of NAC on NLRP3 inflammasome activation in MPP^+^-induced SN4741 cells

**FIGURE 8:**
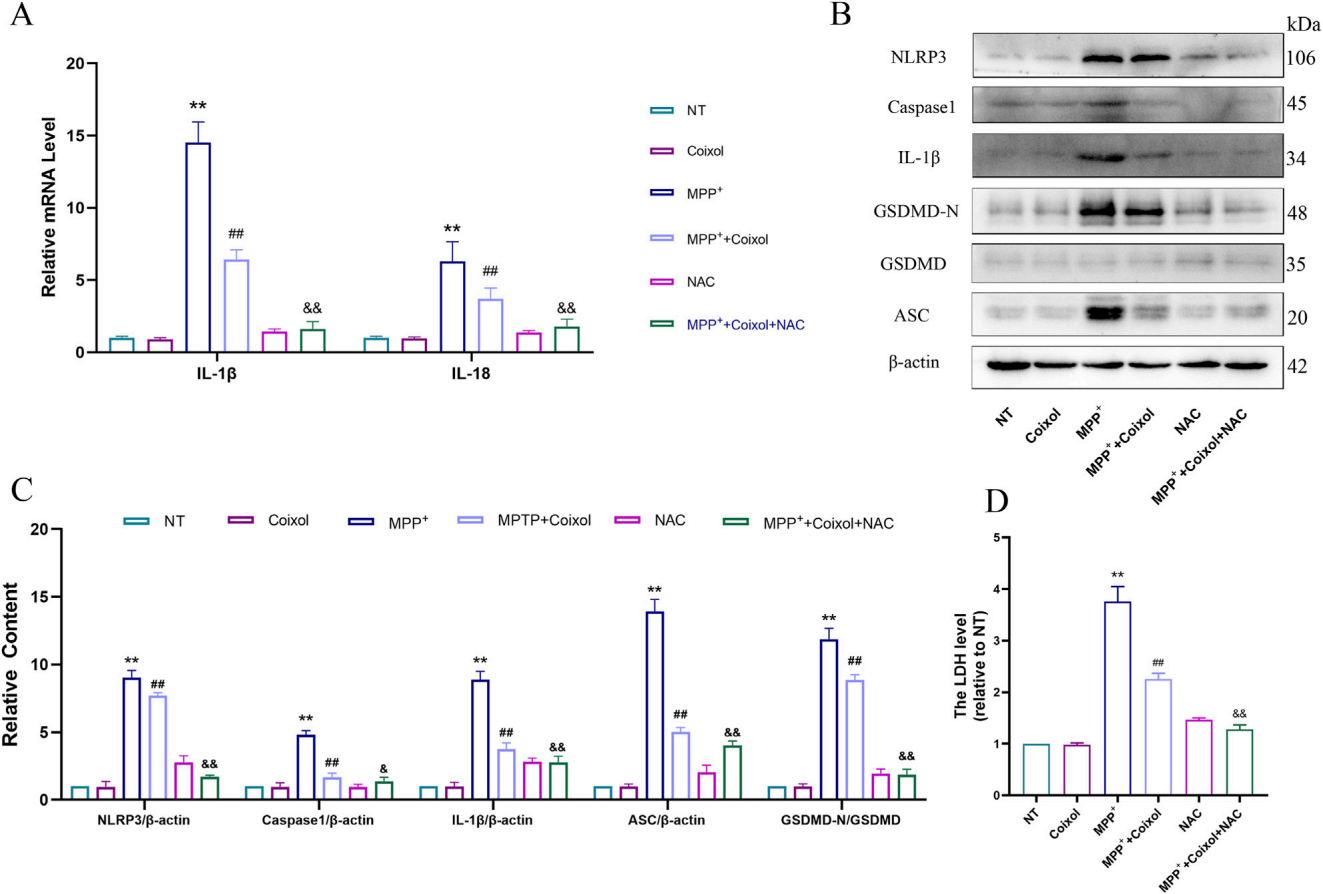
Effect of NAC on NLRP3 inflammasome activation in MPP^+^-induced SN4741 cells. **(A)** The mRNA expressions of IL-1β and IL-18 were detected in SN4741 cells. **(B,C)** The protein levels of NLRP3, Caspase-1, IL-1β, ASC, GSDMD, and GSDMD-N were measured in MPP^+^-induced SN4741 cells using Western blot analysis. **(D)** An LDH assay was conducted to assess the effect of NAC on LDH release. Results are presented as the mean ± SEM (n = 3). ^**^ *p* < *0.01* vs. NT group, ^*##*^ *p* < *0.01* vs. MPP^+^-treated group, and *p* < *0.01* vs. MPP^+^+coixol group.

Studies have shown that excessive mitochondrial ROS production enhances NLRP3 inflammasome activation. NAC (an ROS inhibitor) was used to investigate its effect on NLRP3 inflammasome activation in PD. Our results showed that coixol inhibited the expressions of IL-1β and IL-18, while the expressions of both were further reduced after NAC treatment (Figure 8A). Subsequently, the expressions of NLRP3, Caspase-1, IL-1β, ASC, and GSDMD-N were detected through Western blot analysis in MPP^+^-induced SN4741 cells (Figures 8A–C). The results showed that the expressions of NLRP3, Caspase-1, IL-1β, ASC, and GSDMD-N were decreased in the MPP^+^+coixol+NAC group compared with those in the MPP^+^+coixol group, suggesting that coixol suppressed NLRP3 inflammasome activation. LDH assay results indicated that NAC treatment enhanced the inhibition of coixol on NLRP3 inflammasome activation (Figure 8D). This suggests that downregulation of ROS production can enhance the inhibition of coixol on the NLRP3 inflammasome activation.

## 4 Discussion

PD not only greatly affects the physical and mental health of patients but also imposes a heavy burden on their families. Therefore, it is of great significance to explore the mechanism of PD and find effective prevention and treatment methods. MPTP, a lipophilic compound, can cross the blood–brain barrier and then be rapidly converted into the toxic metabolite MPP^+^ by monoamine oxidase B (MAO-B) (Nicotra and Parvez, 2000). MPP^+^ is selectively absorbed by dopaminergic neurons through the dopamine transporter (DAT), leading to severe mitochondrial respiratory chain dysfunction. MPTP-induced PD mouse models are commonly used as research tools in the study of the pathogenesis of PD (Mustap et al., 2021; Jackson-Lewis and Przedborski, 2007). Our results showed that coixol improved motor dysfunction and neuronal damage in MPTP-injected PD mice by inhibiting neuroinflammation and maintaining mitochondrial function. Our research showed that coixol inhibited neuroinflammation by suppressing overactivated NF-κB and MAPK signaling pathways and regulating NLRP3 inflammasome activation. Moreover, we found that coixol alleviated ROS-triggered mitochondrial damage to suppress NLRP3 inflammasome activation. In the NAC-positive control experiment, it was demonstrated that coixol could inhibit NLRP3 inflammasome overactivation in the inflammatory response and in SN4741 cells triggered by mitochondrial ROS. Overall, our results showed that coixol relieved neurodegeneration in PD mice by inhibiting neuroinflammation and maintaining mitochondrial function.

The research used a behavioral test to investigate the effect of coixol on motor dysfunction in PD mice. PD mice exhibited weight loss, muscle stiffness, and resting tremor symptoms compared with the control group. Coixol treatment improved weight loss and motor dysfunction in MPTP-injected PD mice. In dopaminergic neurons, TH catalyzes the production of the dopamine precursor levodopa (L-DOPA) from tyrosine. TH expression is a hallmark of dopaminergic neurons, and its expression level changes are closely related to the occurrence and development of PD (Huot and Parent, 2007). In the central nervous system, IBA-1, a calcium-binding protein specifically expressed in microglia, participates in regulating the activation of microglia (Norden et al., 2016). In this study, the results of PD mouse brain tissue showed that the expression of TH was decreased, and the expression of IBA-1 was increased. Coixol treatment significantly inhibited the decrease in TH expression and the increase in IBA-1 expression, indicating that coixol prominently protected dopaminergic neurons from activated microglia in the SN. It has been reported that overactivated NLRP3 inflammasome occurred in the SN of PD mice (Zhao et al., 2020). We measured the expressions of NLRP3, Caspase-1, IL-1β, ASC, and GSDMD-N in PD mice. Our results showed that coixol suppressed the activation of NLRP3 and the expressions of IL-1β and IL-18 in PD mice, suggesting that coixol has a neuroprotective effect on PD mice.

The assembly and activation of NLRP3 inflammasome contribute to neuroinflammation, and neuroinflammation is one of the important pathological features of PD. Overactivated NLRP3 inflammasome led to the high expression of NLRP3 inflammasome, inducing neuroinflammation that could accelerate dopaminergic neurodegeneration and result in behavioral dysfunction of PD (Kou et al., 2022). Neuroinflammation is characterized by an increase in activated microglia and astrocytes in the brain, accompanied by the generous production of cytokines, chemokines, ROS, and other substances (Jassim et al., 2021). Extensive research has reported that LPS can initiate neuroinflammation by activating NF-κB and MAPK signaling pathways, which regulate NLR P3 inflammasome activation (Ju et al., 2021; Lo et al., 2022; Lo et al., 2023). In this study, our results showed that coixol treatment suppressed the expression of pro-inflammatory mediators by regulating NF-κB and MAPK signaling pathways and NLRP3 inflammasome activation in BV-2 cells. This study revealed that coixol inhibited neuroinflammation to exert neuroprotection in PD mice.

When mitochondrial function is impaired, the balance between the generation and clearance of oxygen-free radicals is disrupted, with a decrease in SOD activity and an increase in MDA production. Excessive ROS can also damage cellular lipids, proteins, and DNA in neurons, resulting in neuronal degeneration and death (Tu et al., 2019). In this study, we found that coixol enhanced the activity of SOD and CAT and the production of ATP. However, coixol decreased the expression of MDA. It has been reported that AMPK activated the Sirt1/PGC1-α pathway, which protects dopaminergic neurons by promoting mitochondrial biogenesis in the MPTP-induced PD mouse model (Jhuo et al., 2024). MFN1/2 proteins, mainly involved in mitochondrial fusion in mammals, play an important role in mitochondrial biogenesis (Cho et al., 2010). The FUNDC1 protein mediates mitochondrial autophagy in mammalian cells, selectively clears dysfunctional mitochondria, and then reduces the damage of cells and tissues (Chen et al., 2016). Previous studies have demonstrated that improving mitochondrial biogenesis could alleviate dopaminergic neurodegeneration in PD models (Zhang et al., 2023; Hasegawa et al., 2016; Pirooznia et al., 2020). Therefore, we determined the effect of coixol on the Sirt1/PGC1-α/NQO1 signaling pathway and the expressions of MFN1, MFN2, TOM20, and FUNDC1 in MPP^+^-induced SN4741 cells to evaluate mitochondrial damage. Our results showed that coixol activated the Sirt1/SirT5/PGC1-α/NQO1 signaling pathway and increased the expressions of MFN1, MFN2, TOM20, and FUNDC1 in MPP^+^-induced SN4741 cells. The study reveals that coixol significantly inhibits mitochondrial damage in MPP^+^-induced SN4741 cells, suggesting that coixol exerts a neuroprotective effect by activating the Sirt1/SirT5/PGC1- α/NQO1 signaling pathway.

Mitochondrial dysfunction and oxidative stress are key pathological processes in PD (Islam, 2017). However, the interrelationship between mitochondrial dysfunction and neuroinflammation is not yet fully understood. The NLRP3 inflammasome can be activated by various intracellular stimuli, such as extracellular ATP and ROS, which can activate the downstream pathway of the NLRP3 inflammasome to exacerbate the subsequent inflammatory cascade reactions that contribute to pathological changes in PD by regulating the mitochondrial energy metabolism (Khot et al., 2022; Liang et al., 2015; Lu et al., 2014). To investigate the effect of coixol on the NLRP3 inflammasome driven by mitochondrial ROS in SN4741 cells, we used NAC as a positive control. It has been demonstrated that NAC can reduce the generation of intracellular ROS (Zhou et al., 2023). We found that the production of ROS and expressions of NLRP3, ASC, and Caspase-1 were significantly increased in MPP^+^-treated SN4741 cells. This study detected the expressions of inflammatory factors (IL-18 and IL-1β) using PCR and further detected the expression of the NLRP3 pathway-related proteins using Western blot analysis. The results showed that the expression levels of NLRP3, ASC, Caspase-1, GSDMD-N, IL-18, and IL-1β were significantly increased in the MPP^+^-treated group. In the MPP^+^+coixol group, the expression levels of the NLRP3 pathway-related proteins and inflammatory factors were decreased compared with those in the MPP^+^-treated group. In the MPP^+^+coixol+NAC group, the expressions of various proteins and inflammatory factors were further reduced. In addition, the results of the LDH release assay showed that the MPP^+^+coixol group significantly inhibited cell toxicity, and the MPP^+^+coixol+NAC group exhibited even lower LDH release than the MPP^+^+coixol group. These results demonstrate that coixol inhibits NLRP3 inflammasome activation to regulate neuroinflammation by modulating mitochondrial ROS production.

In summary, our results demonstrate that coixol significantly alleviates neuronal pathological damage and mitochondrial damage in MPTP-induced PD mice by downregulating the expression levels of inflammasome-related proteins. The relationship between mitochondrial defects and neuroinflammation remains unclear. This study has investigated the effect of ROS on regulating NLRP3 inflammasome activation in PD mice to elucidate the relevant mechanisms of mitochondrial damage and neuroinflammation. However, the pathological mechanism of PD is complex, and we still need to conduct multi-level and deeper research on PD to explore more related pathways and target drugs.

## Data Availability

The datasets presented in this study can be found in online repositories. The names of the repository/repositories and accession number(s) can be found in the article/Supplementary Material.

## Glossary

ASC: apoptosis-associated speck-like protein containing a CARD
ATP: adenosine triphosphate
Bcl-2: *B-cell leukemia/lymphoma* 2
Bax: Bcl-2-associated X protein
COX-2: cyclooxygenase-2
CAT: catalase
Caspase3: cysteine-requiring aspartate protease 3
Caspase-1: cysteinyl aspartate specific proteinase-1
DAs: dopaminergic neurons
DAT: dopaminergic neurons through the dopamine transporter
DMEM: Dulbecco’s modified Eagle’s medium
DMSO: dimethyl sulfoxide
FUNDC1: fun14 domain-containing protein 1
DAPI: 4′,6-diamidino-2-phenylindole
GSDMD: gasdermin D
GSDMD-N: gasdermin-N domains in GSDMD
GSH-Px: glutathione peroxidase
IL-1β: interleukin-1β
IL-6: interleukin-6
IL-18: interleukin-18
iNOS: inducible nitric oxide synthase
IBA-1: ionized calcium-binding adaptor molecule 1
LDH: lactate dehydrogenase
MFN1: mitofusin 1
MFN2: mitofusin 2
MPTP: 1-methyl-4-phenyl-1,2,3,6-tetrahydropyridine
MAO-B: monoamine oxidase B
MAPK: mitogen-activated protein kinase
NLRP3: NLR family pyrin structural domain 3
NF-κB: nuclear transcription factor κB
NO: nitrogen monoxide
NQO1: NAD (P) H quinone dehydrogenase 1
PD: Parkinson’s disease
PGC-1α: peroxisome proliferator-activated receptor γ coactivator l alpha
ROS: reactive oxygen species
Sirt1: silent information regulator 1
SN: substantia nigra
Str: striatum
TNF-α: tumor necrosis factor-α
TH: tyrosine hydroxylase
TOM20: translocase of outer mitochondrial membrane 20
