# Supplementary material for: Coixol ameliorates dopaminergic neurodegeneration by inhibiting neuroinflammation and protecting mitochondrial function
Source: Front Pharmacol. 2025 Oct 2;16:1657910. doi: 10.3389/fphar.2025.1657910 (PMC12528104; doi:10.3389/fphar.2025.1657910)

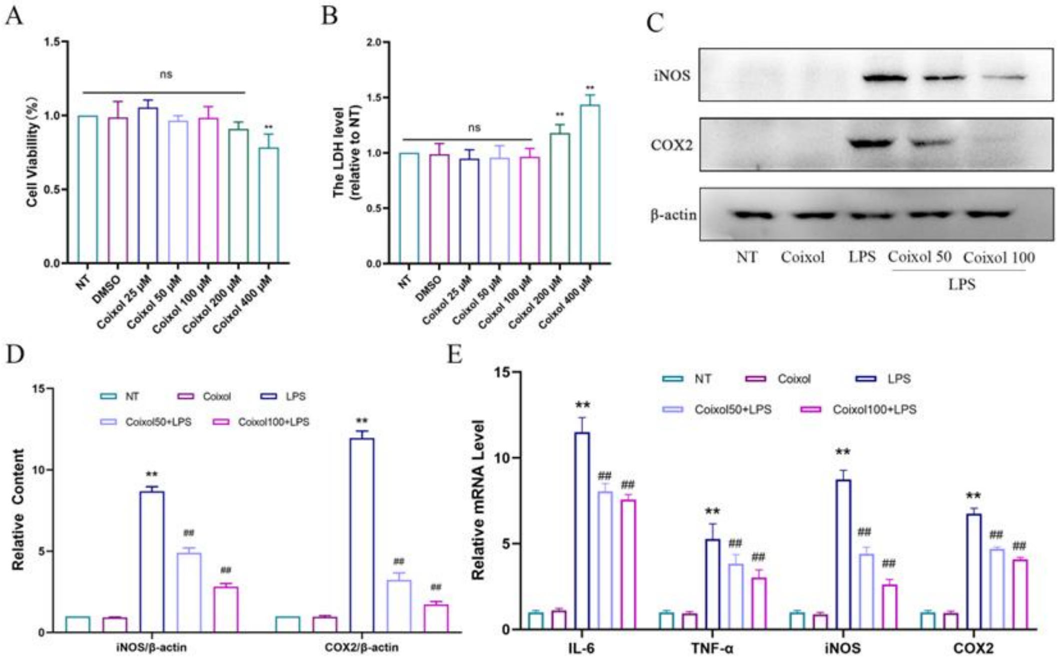

Fig.S1. The effects of Coixol on BV-2 cell viability and Lipopolysaccharide (LPS)-induced inflammatory factors in BV-2 cells. (A) Cell viability (CCK-8) analysis was used to assess the protective effect of Coixol in BV-2 cells. (B) Lactate dehydrogenase release (LDH) assay was conducted to investigate the effect of Coixol on LDH release in LPS-induced BV-2 cells. (C-D) Levels of inducible Nitric Oxide Synthase (iNOS) and cyclooxygenase-2 (COX-2) were measured in LPS-induced BV-2 cells using Western blot. (E) The mRNA expression of Interleukin-6 (IL-6), Tumor Necrosis Factor-alpha (TNF-α), iNOS and COX2 were detected in LPS-induced BV-2 cells. Results are presented as mean ± SEM (n=3). $^{ns}p>0.05$, $^{**}p<0.01$ vs. non-treated (NT) group. $^{##}p<0.01$ vs. LPS-treated group.

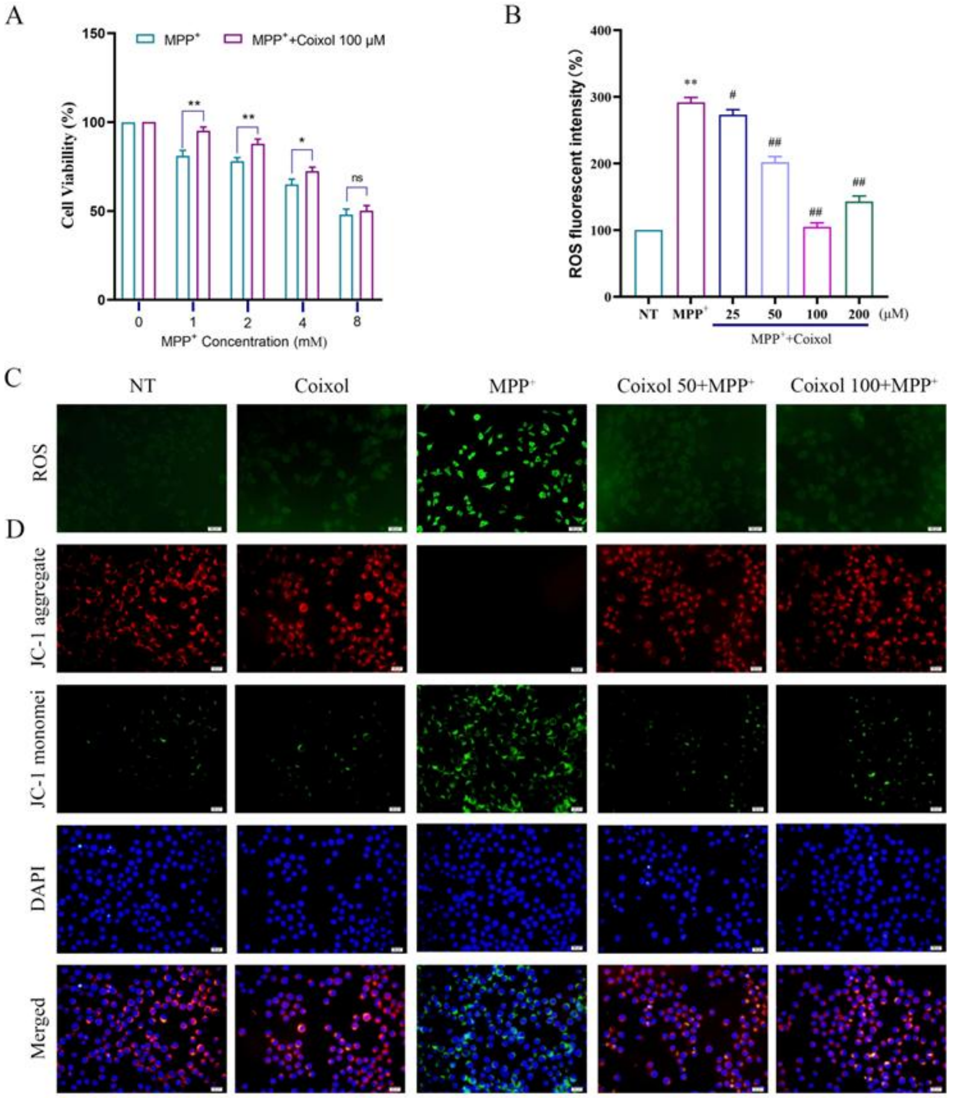

Fig.S2. The effect of Coxiol on mitochondrial damage in MPP+-induced SN4741 cells. (A) CCK-8 analysis was conducted to assess the effect of Coxiol in MPP^+-induced SN4741 cells. (B) Reactive oxygen species (ROS) assay. (C) Mitochondrial membrane potential alterations were evaluated in MPP+-induced SN4741 cells by detecting ROS fluorescence under an inverted microscope (Scale bar=20 μm). (D) Mitochondrial membrane potential alterations were evaluated in MPP+-induced SN4741 cells by investigating JC-1 fluorescence under an inverted microscope (Scale bar=20 μm). Results are presented as mean ± SEM (n=3). $^{**}p<0.01$ vs. NT group. $^{**}p<0.01$, $^{***}p<0.001$, $^{\#}p<0.05$, $^{\#\#}p<0.01$ vs. MPP^+-treated group.

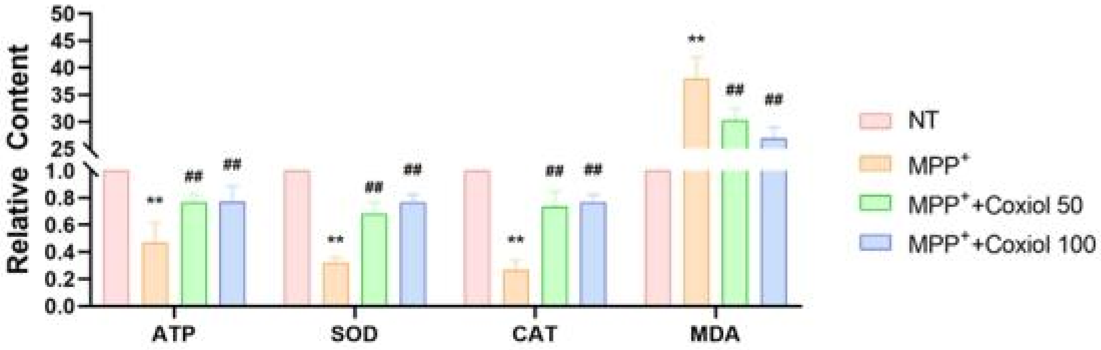

Fig.S3. The effect of Coxiol on MPP+-induced oxidative stress-related indices in SN4741 cells. Adenosine triphosphate (ATP) assay, superoxide dismutase (SOD), Catalase assay (CAT) and Malondialdehyde assay (MDA) were performed on mice midbrain tissues. Results are presented as mean ± SEM (n=3). $^{**}p<0.01$ vs. NT group. $^{\#\#}p<0.01$ vs. MPP+-treated group.

The original gels in Figure 2 and Figure 3.

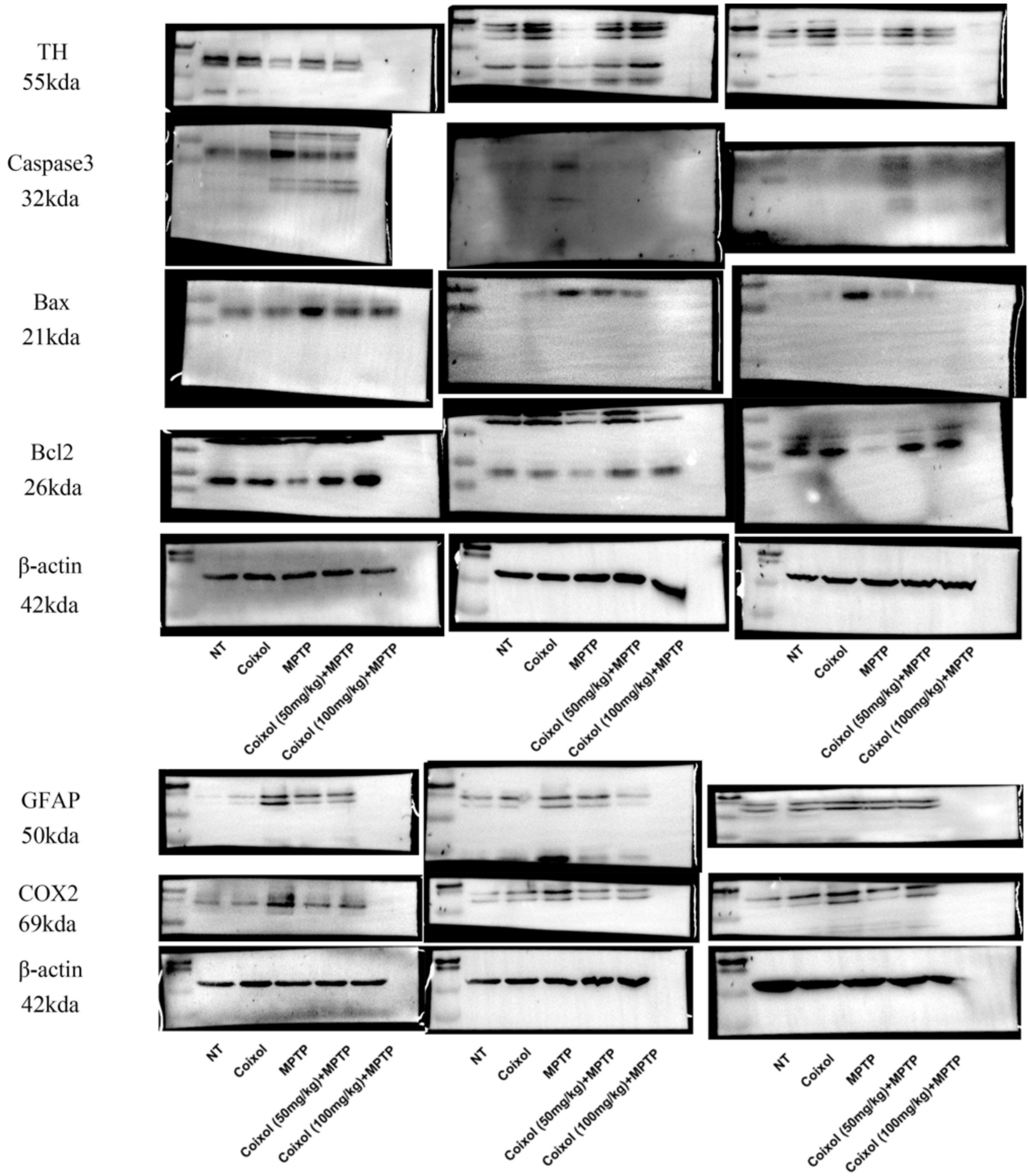

The original gels in Figure 4.

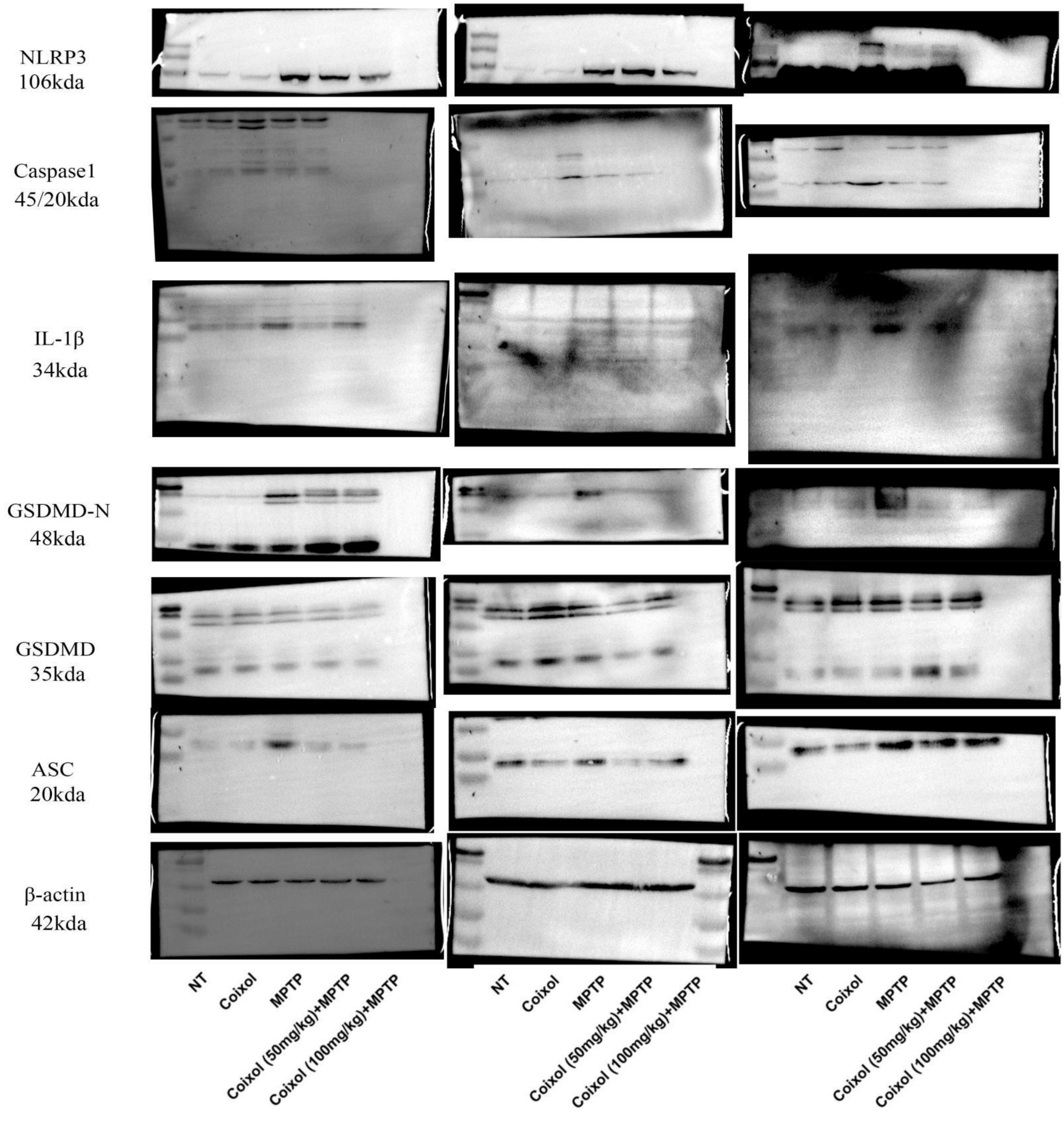

The original gels in Fig.S1 and Figure 5.

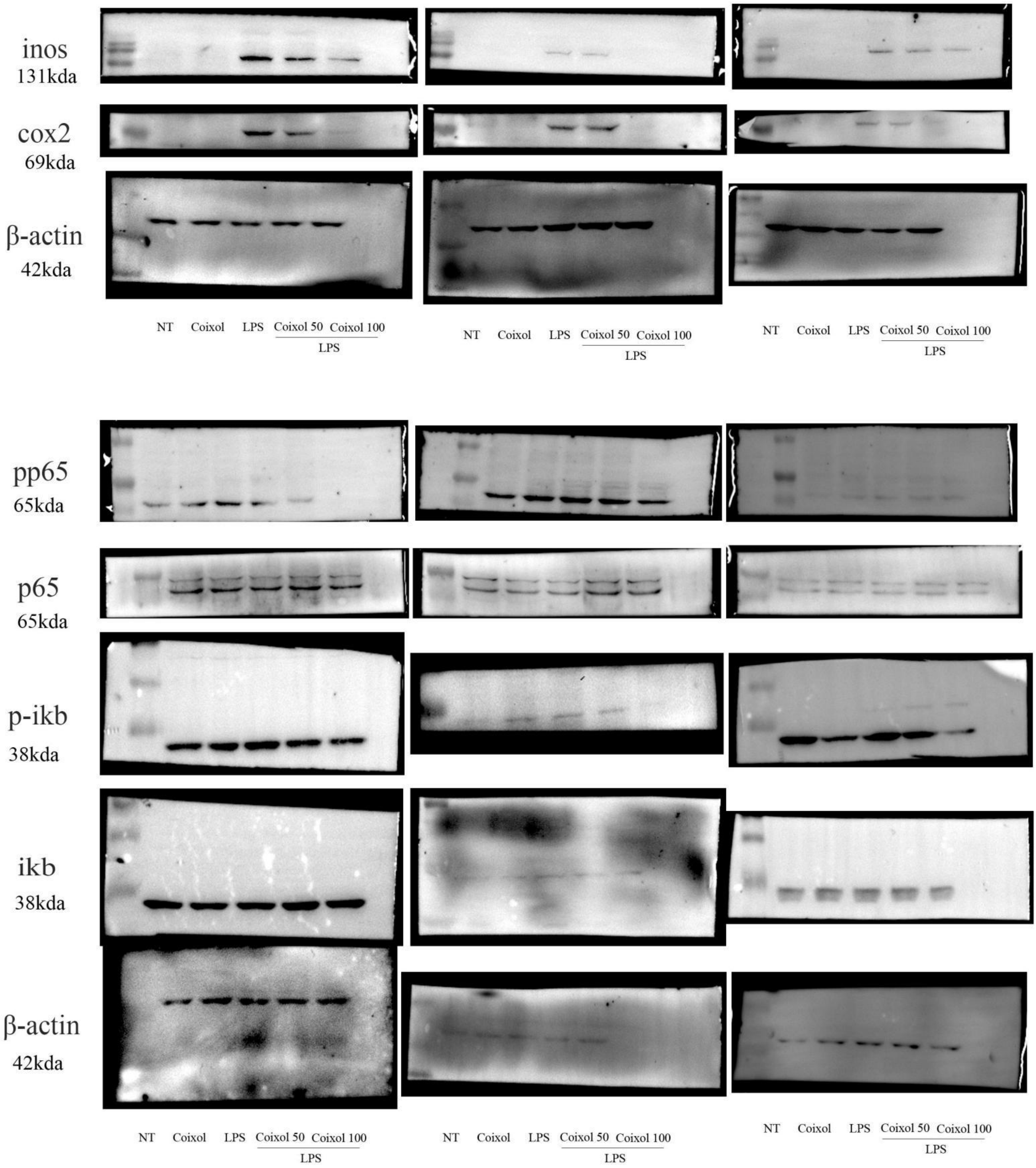

The original gels in Figure 5.

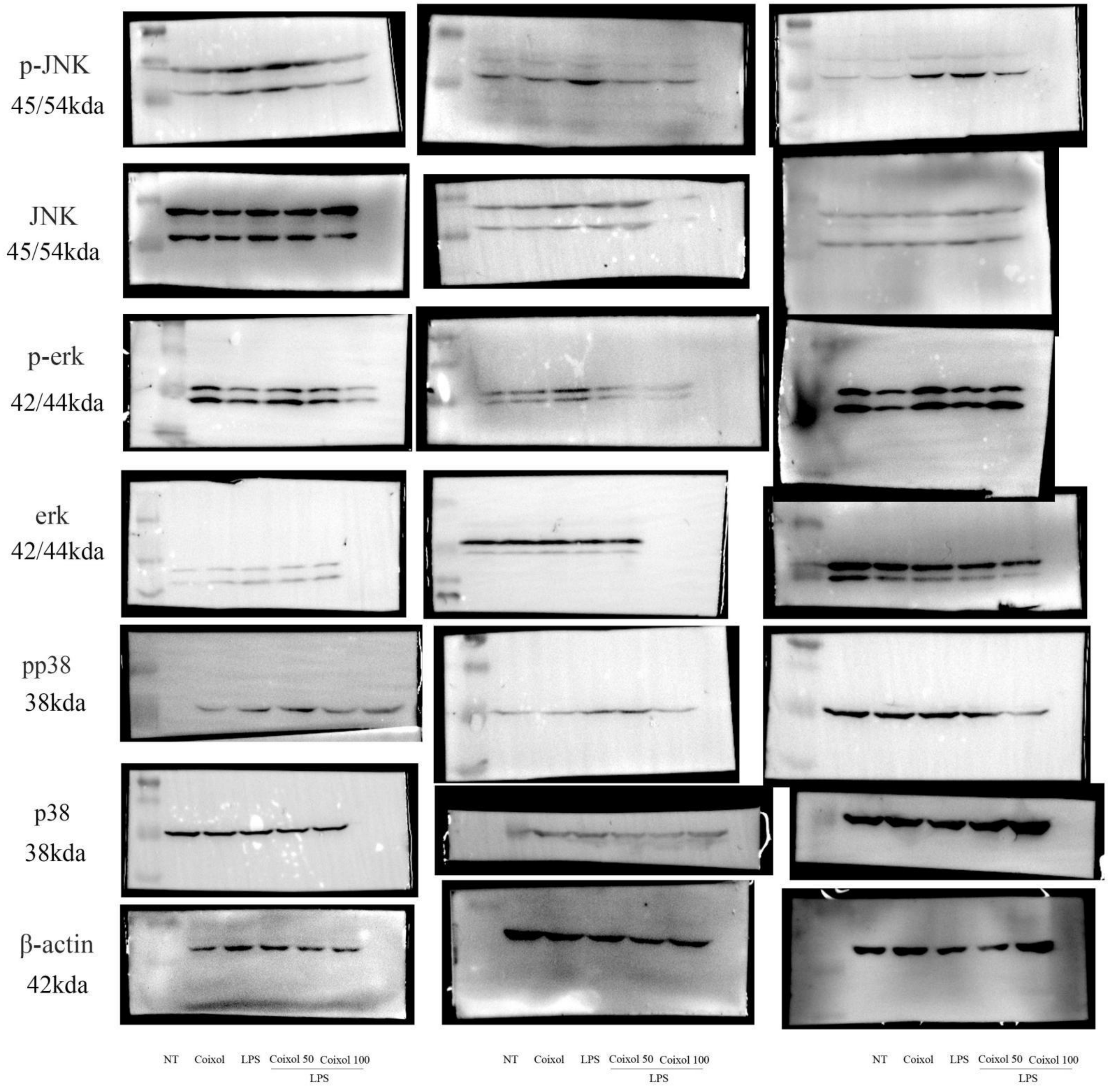

The original gels in Figure 6.

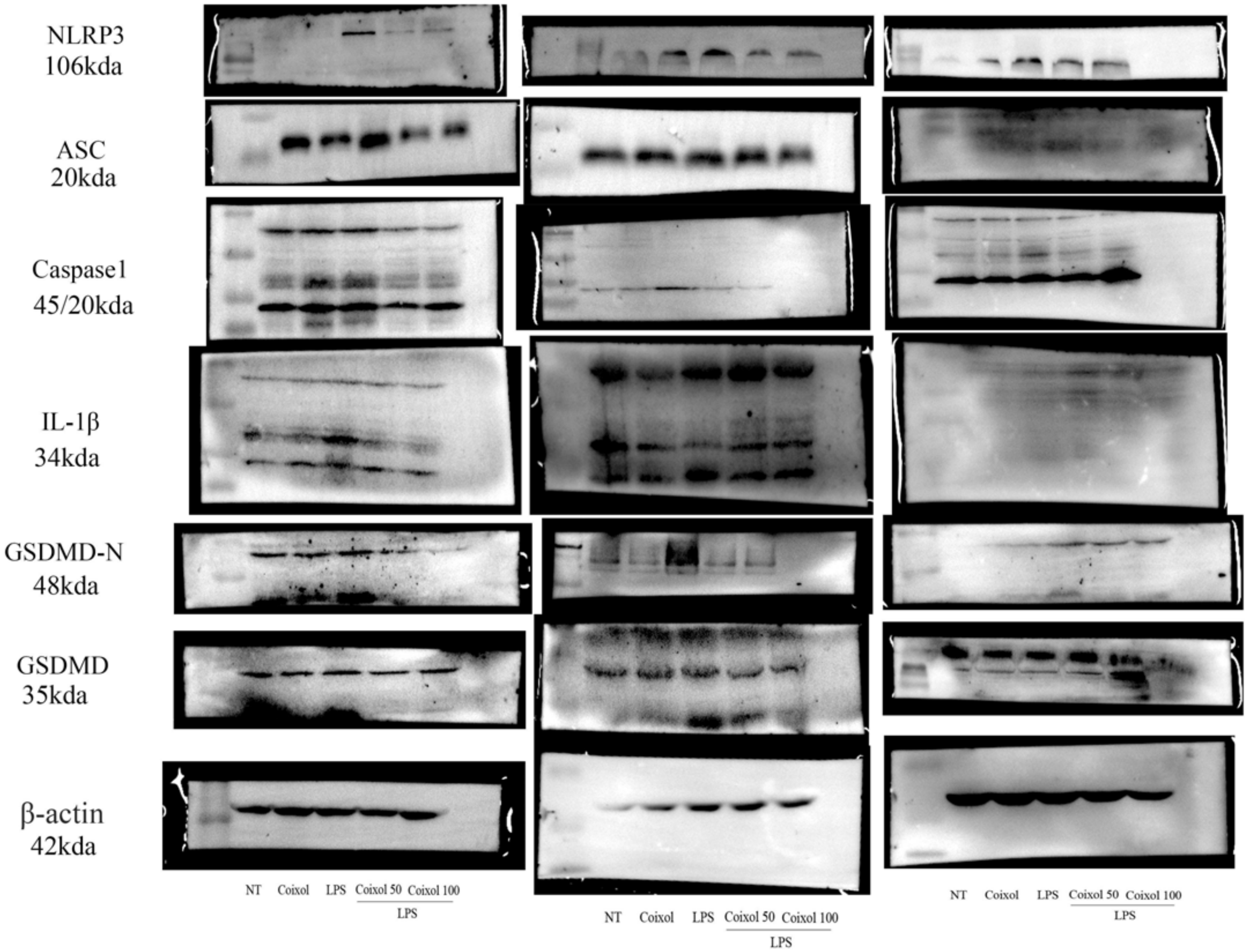

The original gels in Figure 7.

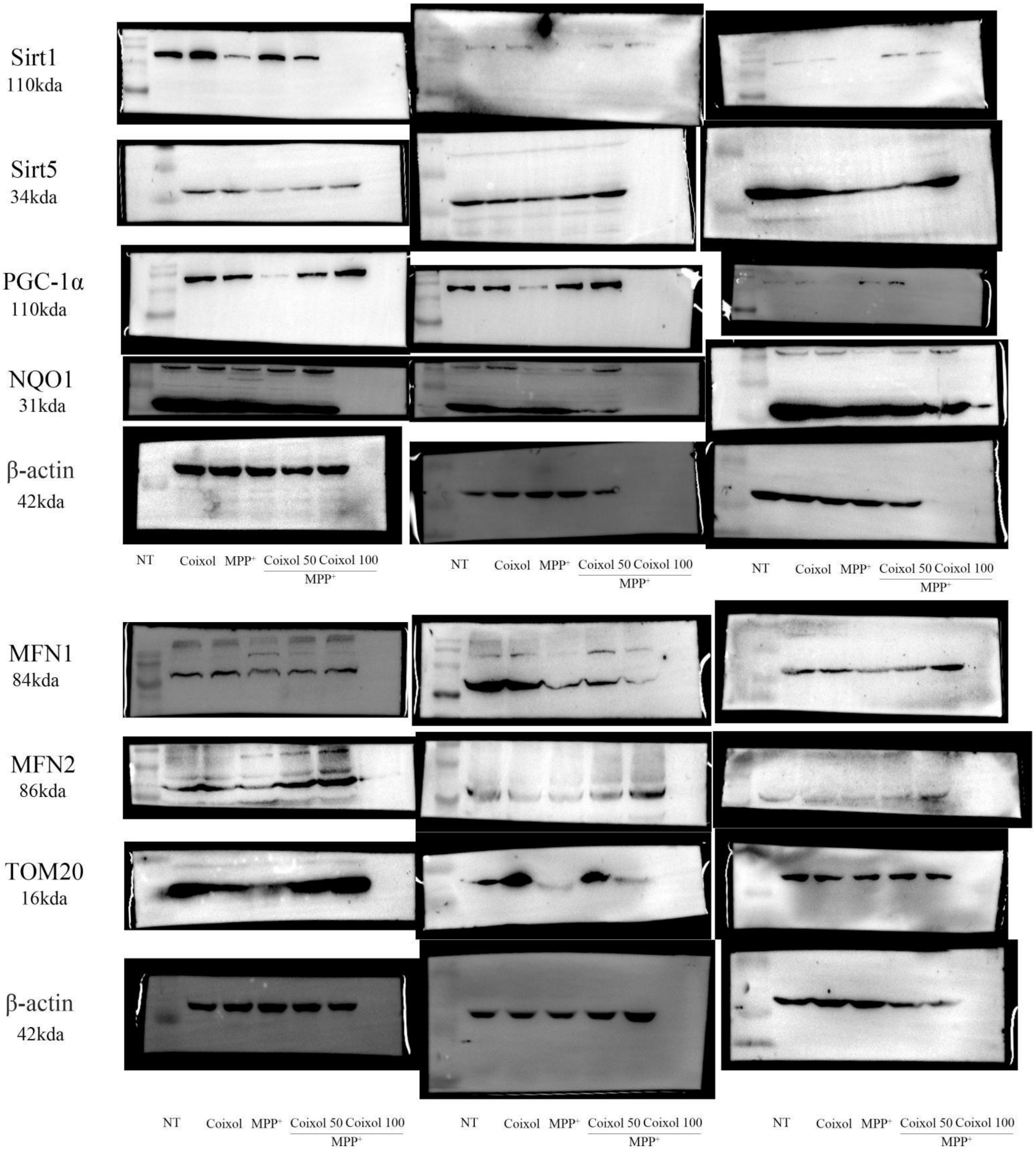

The original gels in Figure 8.

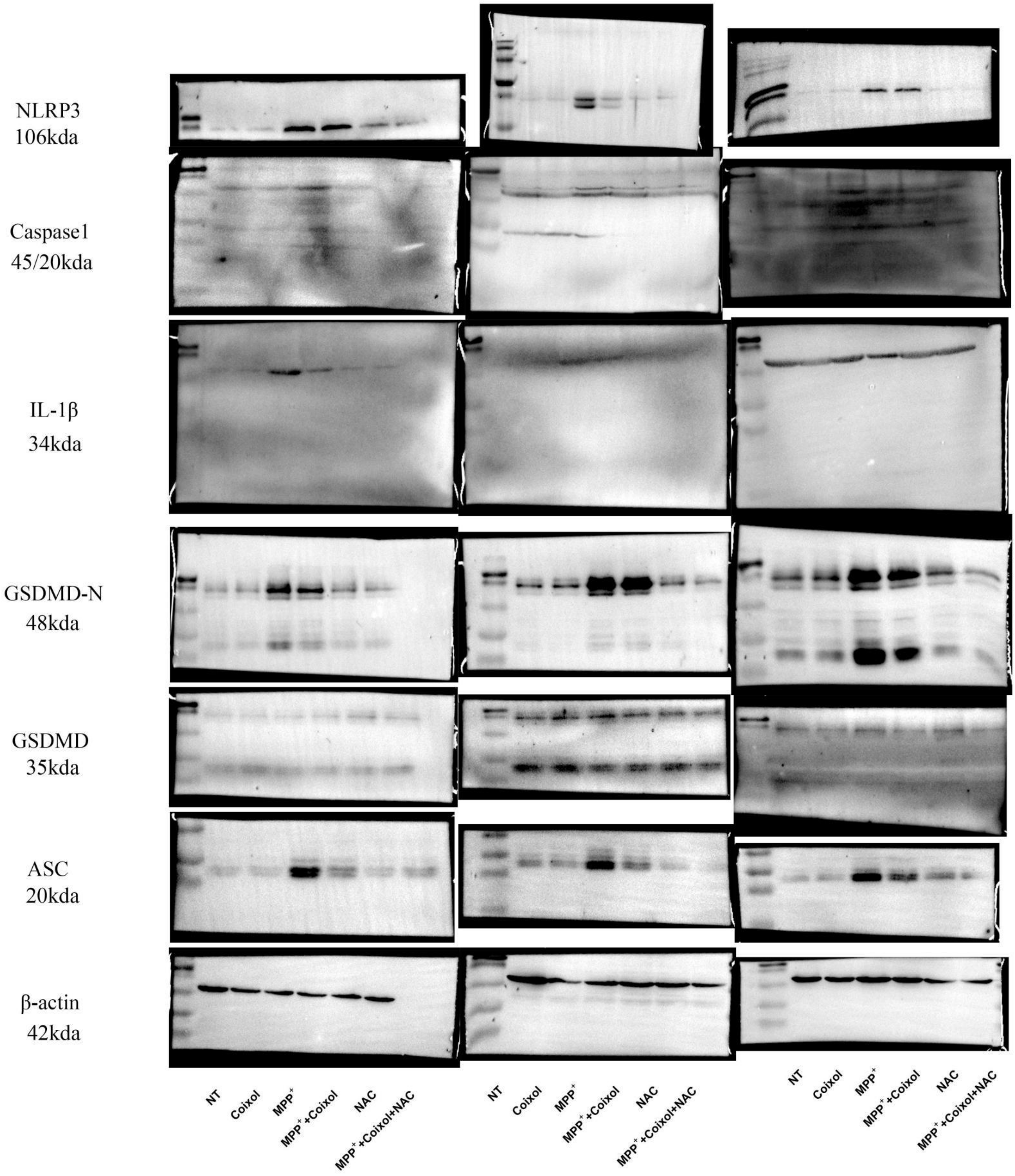

Supplement: Supplementary file 1 [file DataSheet1.pdf]
